# Discovery, Validation and Mechanistic Study of XPO1 Inhibition in the Treatment of Triple-Negative Breast Cancer

**DOI:** 10.3390/cancers16233980

**Published:** 2024-11-27

**Authors:** Amy L. Paulson, Robert F. Gruener, Adam M. Lee, R. Stephanie Huang

**Affiliations:** 1 Department of Molecular Pharmacology and Therapeutics, University of Minnesota School of Medicine, Minneapolis, MN 55455, USA; pauls860@umn.edu; 2 Department of Experimental and Clinical Pharmacology, University of Minnesota School of Pharmacy, Minneapolis, MN 55455, USA; rgruener@umn.edu (R.F.G.); leeam@umn.edu (A.M.L.)

**Keywords:** triple-negative breast cancer (TNBC), selective inhibitors of nuclear export (SINEs), XPO1, RNA-seq, NFKBIA

## Abstract

**Background/Objectives**: Triple-negative breast cancer (TNBC) is an aggressive form of breast cancer with limited treatment options. The nuclear export protein XPO1 has emerged as a potential therapeutic target in cancer, but its role in TNBC has not been fully characterized. This study investigates the potential of repurposing selinexor, an FDA-approved XPO1 inhibitor, as a novel therapeutic options for TNBC. **Methods**: A computational drug repurposing pipeline was used to predict patient tumor responses to hundreds of drugs. We identified XPO1 inhibitors as a candidate drug and validated its efficacy on an independent patient dataset and across various TNBC cell lines. RNA-sequencing after longitudinal XPO1 inhibition and further mechanistic studies were performed to explore and confirm the leading causes of TNBC cell sensitivity to XPO1 inhibition. **Results**: Selinexor significantly reduce the viability of a variety of TNBC cell lines. Mechanistically, selinexor induces TNBC cell death by inhibiting the NF-kB pathway through nuclear retention of NFKBIA. This effect was consistent across multiple TNBC cell lines. **Conclusions**: XPO1 inhibitors show promise as targeted therapies for TNBC patients. New mechanistic insight into the causes leading to TNBC sensitivity to XPO1-inhibition-mediated cell death warrant further clinical trials to evaluate the safety and efficacy in TNBC.

## Simple Summary

Triple-negative breast cancer (TNBC) is an aggressive form of breast cancer with limited treatment options. This study investigates the potential of repurposing selinexor, an XPO1 inhibitor, as a novel therapeutic option for TNBC. By utilizing a computational drug repurposing approach, XPO1 inhibitors were identified to be preferentially sensitive in TNBC compared to other breast cancer subtypes and its efficacy validated in an independent patient dataset and across various TNBC cell lines. Using RNA-sequencing after longitudinal XPO1 inhibition in a panel of TNBC cell lines and Western blotting, we reveal that the XPO1 inhibitor induces TNBC cell death by inhibiting the NF-kB pathway through nuclear retention of NFKBIA, a key regulator of this pathway. These findings suggest that XPO1 inhibitors could be an effective targeted therapy for TNBC and paves the way for more personalized treatment strategies offering hope for better outcomes in TNBC patients.

## 1. Introduction

Breast cancer is the most diagnosed cancer among women with approximately 2.3 million new cases yearly across the globe [1]. Approximately 15% of diagnosed cases present as triple-negative breast cancer (TNBC) and are defined by the lack of estrogen receptors, progesterone receptors, as well as HER2 expression [2,3,4,5]. Compared to other breast cancer subtypes, TNBC is characteristically more aggressive, associated with higher mortality rates, metastatic potential, rate of relapse and poorer prognosis [6]. Although demonstrating low effectiveness and a high incidence of side effects, cytotoxic chemotherapy remains to be the chief systemic treatment for TNBC. Therefore, there is an urgent need to identify effective therapeutic options for TNBC patients to combat these aggressive diseases.

Conventional methods to develop new drugs for cancer treatment often involve target identification, compound screens and optimization, preclinical and clinical evaluation and FDA approval. This process can be extremely time-consuming and costly with only a small percentage of drugs actually receiving market approval [7,8,9]. In contrast, drug repurposing, a strategy for identifying new indications for already-approved drugs, can offer new treatment opportunities for a specific group of interest while also reducing the cost and time of initial research and development. Our group previously established a computational approach to accurately predict patient tumor response to hundreds of medications based on the use of large-scale high-throughput in vitro drug screening data and patient tumor transcriptomic information [10,11,12]. This approach has enabled us to virtually screen patients against hundreds of compounds and generate hypotheses about which drugs can be repurposed for new indications. In this study, we sought to identify an efficacious therapy to repurpose for the treatment of TNBC. Through our computational process, we nominated selinexor, an XPO1 inhibitor approved to treat multiple myeloma (MM) for TNBC.

XPO1 is one of several essential nuclear export proteins that mediate the nuclear-cytoplasmic transport of proteins (>40 kDa) and multiple RNA species through a nuclear pore complex [13]. XPO1 recognizes cargo species for export from the nucleus by the presence of a nuclear export signal (NES) sequence on a target protein. Many cancers present with an elevated expression of XPO1, such as multiple myeloma, glioma, lung and breast [14,15]. There have been more than 200 reported cell regulatory proteins that XPO1 is solely responsible for exporting—including multiple tumor suppressing proteins (TSPs) such as NFKBIA, p21, p53 and pRb [13,16,17,18]. The correct subcellular distribution of TSPs often plays a critical role in determining normal cell function or tumorigenesis and cellular proliferation [19]. Thus far, selinexor-induced cellular apoptosis and growth inhibition appears to be cancer-context-dependent, with various TSPs being implicated in different cancer contexts [17,20]. Therefore, we set out to explore the mechanism of action for selinexor sensitivity in TNBC with the goal to elucidate markers of response, guide clinical trial recommendation and facilitate the proposal of synergistic combination therapies.

Taken together, we applied a computational drug repurposing pipeline towards repurposing efficacious drugs to treat TNBC. We then experimentally validated the predicted efficacy of XPO1 inhibitors using a broad panel of TNBC cell lines. Importantly, we examined the mechanism of TNBC selinexor sensitivity by conducting a longitudinal RNA-sequencing (RNA-seq) experiment following selinexor exposure on a collection of TNBC cell lines. NF-kB pathway was found to be a critical mediator of selinexor response. Further genetic perturbation of the NF-kB pathway indicated that NFKBIA is necessary for sensitivity to selinexor.

## 2. Materials and Methods

### 2.1. Data Acquisition

The Molecular Taxonomy of Breast Cancer International Consortium (METABRIC) study [21], as well as The Cancer Genome Atlas (TCGA) breast cancer dataset [22], were downloaded from cBioportal [23]. Prediction models were constructed from combining The Cancer Therapeutics Response Portal Version 2 (CTRPv2) [24] cancer cell line drug response database with the cancer cell line gene expression data from the Broad Institute’s Cancer Cell Line Encyclopedia (CCLE) [25].

### 2.2. Constructing Models and Predicting Drug Sensitivities for TNBC Patients

Methods for imputing drug response in METABRIC and TCGA breast cancer patients using the CTRP/CCLE cell line data were carried out using the R package OncoPredict (RRID:SCR_023872) and were based on those previously described in [10]. Since the METABRIC dataset was microarray, Illumina probes were mapped to gene names, genes with multiple probes were averaged if they had a correlation value greater than 0.6, and NA values were excluded. For both METABRIC and TCGA, genes were filtered to common genes between patient data and CCLE cell line data and then homogenized using ComBat (RRID:SCR_010974) to account for batch effects between the two sources [26]. Feature selection was then performed by removing 20% of genes with the lowest variation in gene expression across all samples. After power transforming AUC values from CTRP, a linear ridge regression model was fit for each drug independently. Once all models were fit, we inputted the homogenized gene expression data between patients and cell lines into each model and obtained imputed drug sensitivity scores for each patient to every drug in the CTRP. To predict compounds that were more sensitive in TNBC patients compared to other subtypes, tumors in each study were grouped by PAM50 status (basal or non-basal) or receptor status (receptor positive or negative) and Student *t*-tests were performed on the imputed drug sensitivity data for each compound between the two groups. A multiple comparison correction was carried out using the Bonferroni method.

### 2.3. Cell Culture

Human TNBC cell lines BT-549 (RRID:CVCL_1092), HCC-38 (RRID:CVCL_1267), HCC-1806 (RRID:CVCL_1258), MDA-MB-157 (RRID:CVCL_0618), MDA-MB-231 (RRID:CVCL_0062) and MDA-MB-468 (RRID:CVCL_0419) were purchased from the American Type Culture Collection (ATCC, Manassas, VA, USA). Additionally, CAL-120 (RRID:CVCL_1104) was purchased from the Leibniz Institute DSMZ (Braunschweig, Germany). Cell lines were cultured in RPMI-1640 supplemented with 10% FBS at 37 °C and 5% CO_2_ atmosphere. Cell lines were selected to represent a diverse array of TNBC subtypes. All cell lines were screened and tested negative for mycoplasma using the Universal Mycoplasma Detection Kit protocol (ATCC, Manassas, VA, USA).

### 2.4. Lentivirus-Mediated Gene Transduction

shRNA knockdown versions of NFKBIA or control scramble in BT-549 and HCC-1806 were generated using lentivirus, which was designed using VectorBuilder Inc. (Chicago, IL, USA). Successfully transduced cells were GFP-labeled and selection was carried out via puromycin.

### 2.5. Drug Preparation

Selinexor was obtained from Selleck Chemicals (Houston, TX, USA), reconstituted in DMSO and aliquoted into 10 mM stocks stored at −80 °C. Medium was used to dilute selinexor to its final concentration and a DMSO of no greater than 0.1% volume was used for all experiments.

### 2.6. Cellular Viability and Apoptosis Assays

Cellular viability following selinexor treatment was measured using the cellular proliferation reagent WST-1 (Cell-Pro-Ro; Roche Applied Science, Indianapolis, IN, USA) following the manufacturer’s protocol. Cellular growth inhibition was measured 72 h post drug treatment by incubation with WST-1 for 2 h and absorbance was measured at a wavelength of 450 nm on a Synergy HTX Multi-Mode Plate reader (BioTek Instruments, Winooski, VT, USA). Cellular viability was determined by normalizing raw absorbance readings to the average values of the control treatment condition on each plate and IC50 values were derived using GraphPad Prism 10 version 10.4.0. 

CellEventTM Caspase-3/7 Green Detection Reagent (Invitrogen, Life Technologies, Carlsbad, CA, USA) was used to assess cellular apoptosis according to the protocol for 72 h post drug treatment. Cells were Hoechst-stained (Thermo Scientific, Waltham, MA, USA) and imaged using a CytationTM 1 Cell Imaging Multi-Mode Reader (BioTek Instruments, Winooski, VT, USA) every 6 h in the DAPI, GFP and Brightfield channels. Automatic background flattening parameters were used to remove the background fluorescence from the GFP and DAPI channels and object masking thresholds were set to identify each cell for counting. DAPI fluorescence was used for identifying the total cell count and a subpopulation count of GFP fluorescence was used to identify apoptotic cells. 

All results are reported as a mean and standard deviation of three independent biological replicates, each containing a minimum of three technical replicates for each treatment condition.

### 2.7. RNA Sequencing and Gene Expression Analysis

BT-549 (basal-like, BL1), CAL-120 (basal-like, BL1), HCC-1806 (basal-like, BL2), and MDA-MB-468 (mesenchymal, MSL) cells were treated with DMSO control or 200 nM selinexor. The selinexor concentration was chosen to be slightly higher than the average IC50 across all cell lines previously determined by the cellular viability assay. Samples were collected at 8 and 24 h post treatment and total RNA was isolated and purified using Quick-RNATM Miniprep Plus Kit (ZYMO Research, Irvine, CA, USA) following the manufacturer’s instructions. All samples were processed at the UMN Genomics Center (RRID:SCR_012413) for library preparation and sequencing at a read depth of 20 million reads/sample. FASTQ files were trimmed using Trimmomatic (RRID:SCR_011848), aligned to the transcriptome using HISAT2 (RRID:SCR_015530), sorted with SAMTOOLS (RRID:SCR_002105), and gene counts were obtained with featureCounts (RRID:SCR_012919). Differentially expressed genes between DMSO and drug-treated samples for each time point were identified using DESeq2 (RRID:SCR_015687). Pathways of interested significantly associated with differentially expressed genes were identified using ReactomeGSA (RRID:SCR_003485) and Gene Set Enrichment Analysis (GSEA) (RRID:SCR_003199). For ReactomeGSA, the weighted gene set analysis PADOG method was selected to weigh genes based on their prevalence in different pathways. Statistical significance was evaluated at α = 0.05 unless otherwise corrected for multiple comparisons.

### 2.8. qPCR

Total RNA was extracted using Quick-RNATM Miniprep Plus Kit (ZYMO Research, Irvine, CA, USA) following the manufacturer’s instructions. cDNA was prepared using applied biosystems High-Capacity cDNA Reverse Transcription Kit and quantitative PCR assays were conducted on an Applied Biosystems 7500 Real-Time PCR System. The expression of *NFKBIA* was normalized to the housekeeping gene *HPRT1*.

### 2.9. Western Blotting

Whole-cell lysates of BT-549, HCC-1806, and MBA-MB-468 were collected using RIPA Lysis and Extraction Buffer (Thermo Scientific, Waltham, MA, USA) with protease and phosphatase inhibitor (Thermo Scientific, Waltham, MA, USA) dissolved within. Nuclear and cytoplasmic fractions of cells utilized within experiments was collected using NE-PER Nuclear and Cytoplasmic Extraction Reagent Kit (Thermo Scientific) and isolated according to the manufacturer’s protocol. In experiments involving selinexor, cells were treated with 200 nM selinexor for 24 h. 20 µg of total protein was loaded per lane onto a 4–20% Mini-PROTEAN^®^ TGXTM Precast Protein Gel (BIO-RAD, Hercules, CA, USA) and electrophoresis was run at 180 volts for 40 min. Dry transfer onto a PVDF membrane was then performed using an iBlotTM 2 transfer system and its corresponding transfer stacks (Thermo Scientific, Waltham, MA, USA). The antibodies used were NFKBIA (Abcam Cat# ab32518, RRID:AB_733068), XPO1 (Cell Signaling Technology Cat# 46249, RRID:AB_2799298), *β*-actin as a loading control, (Cell Signaling Technology Cat# 3700, RRID:AB_2242334), and HDAC1 as a nuclear control (ABclonal Cat# A19571, RRID:AB_2862675). The imaging of blots was conducted using horseradish peroxidase-conjugated secondary antibodies on a LI-COR Odyssey Fc imager. Image processing was analyzed with ImageJ version 1.53 (RRID:SCR_003070). Statistical significance was evaluated at α = 0.05. Raw images of all Western blots contained in this manuscript can be found in the Supplemental File titled Westerns.

### 2.10. Immunofluorescence Staining

BT-549, CAL-120, HCC-1806, and MDA-MB-468 cell lines were utilized for immunofluorescence staining. Cells were seeded on a 24-well BioCoat plate (Corning #354414), allowed to adhere, and then treated with 200 nM selinexor or DMSO control for 24 h. Cells were fixed with 4% PFA (Electron Microscopy Sciences, Hatfield, PA, USA), permeabilized with 0.2% Triton^®^ X-100 (Millipore Sigma #9002-93-1), and blocked with 2% BSA in PBST. Cells were incubated in primary antibody against NFKBIA (Abcam Cat# ab32518, RRID:AB_733068) overnight at 4 °C with gentle shaking while secondary antibody (Abcam Cat# ab150077, RRID:AB_2630356) incubation was for 1 h at room temperature. Cells were then Hoechst-stained and imaged on a CytationTM 1 Cell Imaging Multi-Mode Reader (BioTek Instruments, Winooski, VT, USA). Between each step, cells were washed with PBST multiple times. Automatic background flattening parameters were used to remove background fluorescence from the GFP and DAPI channels and object-masking thresholds were set to identify each cell for counting. DAPI fluorescence was used for identifying the total cell count and a subpopulation count of GFP fluorescence was used to identify the NFKBIA abundance within the nucleus. A percentage of GFP fluorescence within the nucleus compared to total GFP fluorescence was calculated and compared between drug treated and control samples to determine the significance of NFKBIA nuclear localization. Statistical significance was evaluated at α = 0.05, unless otherwise corrected for multiple comparisons. 

### 2.11. Role of Funders

This study was supported by NIH/NCI Grants R01CA204856 (R.S.H) and a University of Minnesota (UMN) Office of Academic Clinical Affairs (OACA) Grant-in-Aid Program (GIA) award. R.S.H. also received support from NIH/NCI R01CA229618, UMN OACA Faculty Research Development grant, a UMN Masonic Cancer Center CRTI Exceptional Translational Research award, and UMN College of Pharmacy SURRGE award. The funders did not influence the research discovery made in this study.

## 3. Results

### 3.1. XPO1 Inhibitor Is Predicted to Be More Sensitive in TNBC Patient Tumors Compared to Other Breast Cancer Subtypes

With the goal of identifying a new effective therapy for TNBC patients, we utilized the CTRP’s unique compound screen, consisting of 496 compounds across 887 cancer cell lines and used corresponding RNA-Seq gene expression data from CCLE to build a ridge regression model as described in the methods. Drug response models were applied to TCGA breast cancer (discovery) and METABRIC (validation) tumor transcriptome profiles to obtain an imputed drug sensitivity score for each drug against each patient tumor. The drug response models enable us to translate the cell line drug response to patients based on transcriptomic information and look for drugs that are predicted to be particularly effective in TNBC. We performed this analysis in two independent breast cancer cohorts to ensure robustness and the increased odds of successfully nominating a candidate drug.

**Figure 1:**
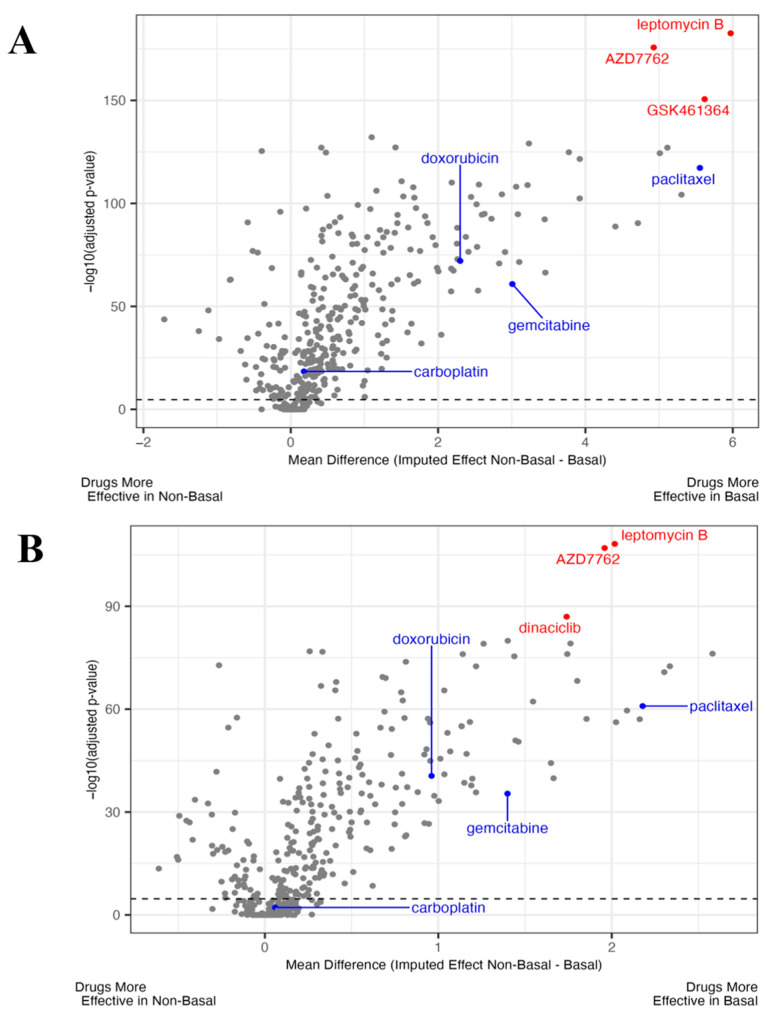
XPO1 inhibitor was predicted to be more effective in METABRIC and TCGA basal type breast cancer patients. (**A**) Using our novel virtual drug screening pipeline, we imputed the drug response in the METABRIC breast cancer cohort and stratified patients into TNBC and non-TNBC (i.e., basal or non-basal). Plotted are the *t*-test results from the comparison of a drug’s predicted effect in TNBC vs. non-TNBC patients, with the effect size shown on the *x* axis and significance (−log10 of adjusted *p* -value based on a number of test correction using a Bonferroni adjustment) on the *y* axis. Dashed black line representing Bonferroni *p*-value of −log10(0.01). A red dot represents one of the top 3 drugs predicted to be the most efficacious in TNBC patient tumors. The blue dots highlight the current standard-of-care treatment for TNBC patients. The PAM50 status was used due to incomplete IHC status within clinical information. (**B**) The same analysis as (**A**) was performed in the independent TCGA clinical breast cancer dataset. In both, leptomycin B, an XPO1 inhibitor is the most significant compound and has one of the largest effect sizes showing higher predicted sensitivity in basal populations.

To identify drugs that are more effective in TNBC patient populations, patients were stratified based on their PAM50 status (non-basal or basal) for METABRIC and TCGA, and patterns of imputed drug sensitivity by subtype were compared. PAM50 basal status was used as a proxy for clinical TNBC given their shared biology and since clinical receptor status was not available for all patients. Student *t*-test results comparing a drug’s predicted sensitivity score for non-basal compared to basal statuses in the METABRIC and in TCGA breast cancer patients are shown in Figure 1A and Figure 1B, respectively, for all 427 drugs imputed. For both METABRIC and TCGA, many of these drugs are significant at a nominal Bonferroni adjusted *p*-value of 0.01 due to the large sample size of breast cancer patients (*n* = 1975 and *n* = 1085, respectively). Therefore, we chose to focus on the drugs with the highest effect size and significance. When looking at this, we found consistent results between the two independent patient datasets. Of the top 10 compounds from each analysis, five were present in both. Among them, leptomycin B (an XPO1 inhibitor) appeared as the most significant result in both the METABRIC and TCGA datasets. Additionally, when compared to current standard-of-care treatments for TNBC, our imputed sensitivity scores for each show preferential sensitivity toward TNBC but are lower than leptomycin B in terms of both effect size and significance. Also, the FDA recently approved an XPO1 inhibitor (selinexor) for MM, providing an additional rationale to focus on further developing/repurposing XPO1 inhibitors for the treatment of TNBC.

### 3.2. In Vitro Screening of Selinexor Validates XPO1 Inhibitor Sensitivity Across a Wide Array of TNBC Cell Lines and Subtypes

**Figure 2:**
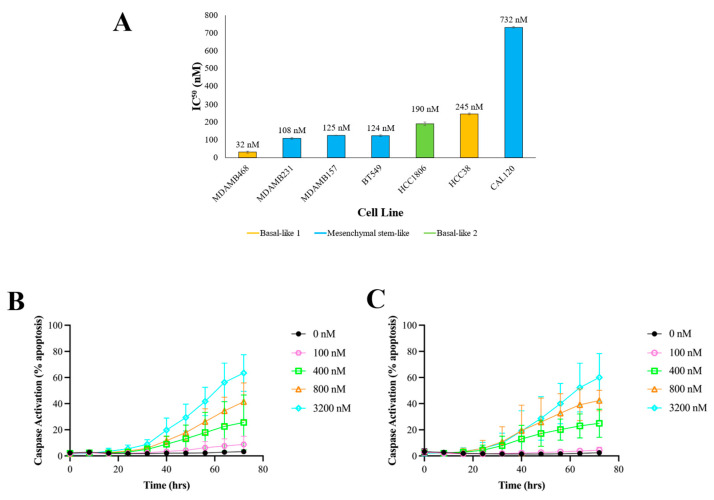
In vitro screening validates selinexor sensitivity across a multitude of TNBC subtypes. (**A**) IC50 values for MDA-MB-468, MDA-MB-231, MDA-MB-157, BT-549, HCC1-806, HCC-38 and CAL-120, each of which are colored by their TNBC subtype classified by gene profiling. A 10-point dose–response curve was fit to viability data from these cell lines after 72 h treatment with between 0 and 3200 nM selinexor. IC50 values with standard error bars factoring in biological replicates are shown. (**B**) Percent apoptosis after various selinexor exposure overtime in BT-549 cells. Caspase activation was measured via GFP fluorescence every 8 h following drug treatment for a total of 72 h. (**C**) Percent apoptosis after various degrees of selinexor exposure overtime in MDA-MB-231 cells. Two-way ANOVA was performed for both panels (**B**,**C**). Statistically significant differences were found among treatment groups at a *p*-value less than 0.05.

To confirm XPO1 inhibitors’ sensitivity for the treatment of TNBC, we tested the efficacy of selinexor in a collection of TNBC cell lines from a variety of TNBC subtypes. Cell lines were exposed to increasing concentrations of selinexor, and cell growth inhibition was measured as described in the Methods section. All seven TNBC cancer cell lines showed significant growth inhibition following selinexor treatment with IC50 values ranging from 32 nM to 732 nM (Figure 2A). As a point of reference, the Cmax of selinexor observed in the clinical trials leading to the drug’s approval in MM was reported to be 680 ng/mL (1533.9 nM) [27]. All TNBC cell lines evaluated were well below this and suggest that selinexor is efficacious in killing TNBC cells for the most part at a rate comparable to, if not better than, those reported previously in initial MM screenings. This finding was also supported by our investigation of other XPO1 inhibitors screened in the CTRP (Supplemental Figure S1).

In addition to cell growth inhibition, cellular apoptosis was assessed using CellEventTM Caspase-3/7 following selinexor treatment in two TNBC cell lines, namely BT-549 (Figure 2B) and MDA-MB-231 (Figure 2C). Dose-dependent apoptosis was observed in both cell lines which occurred starting at approximately 24 h after selinexor treatment. Both cell lines displayed statistically significant differences between the control and drug treated at a *p*-value less than 0.05 when assessed with a two-way ANOVA. Taken together, these experiments demonstrate selinexor’s efficacy in inhibiting TNBC cellular growth via apoptosis-mediated cell death.

### 3.3. XPO1 Expression Alone Does Not Explain TNBC Cell Line Sensitivity to Selinexor

**Figure 3:**
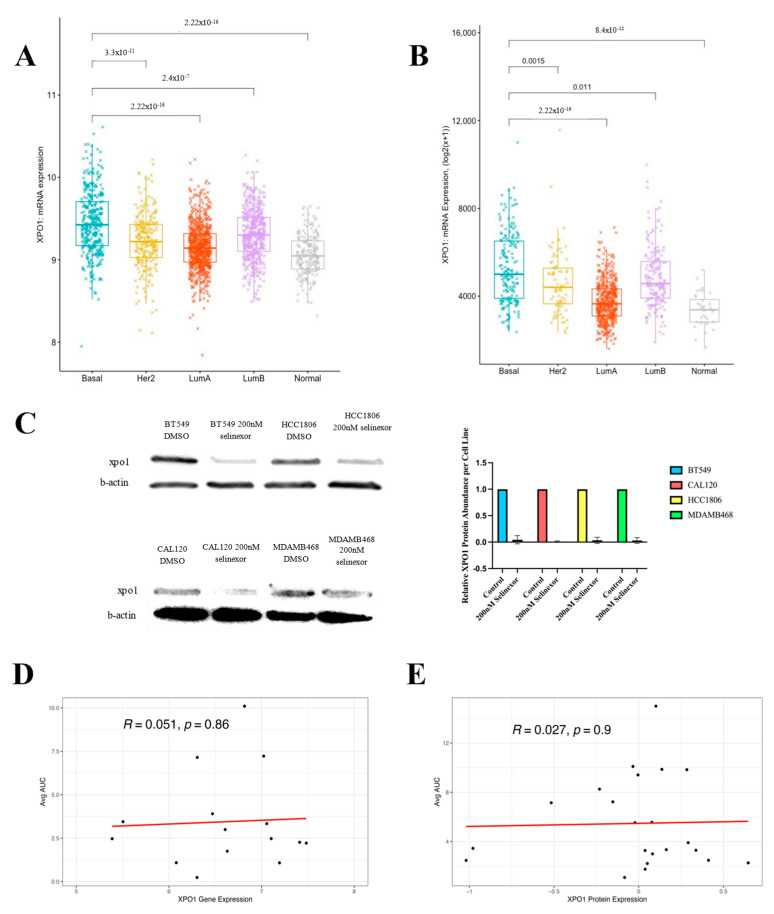
XPO1 expression alone does not explain TNBC sensitivity to selinexor. (**A**) mRNA expression levels of *XPO1* obtained by microarray in patients from the METABRIC dataset categorized by breast cancer subtype. Student *t*-test *p*-value significance between basal and other TNBC subtypes are displayed in brackets above the subtypes. (**B**) mRNA expression levels of *XPO1* obtained by RNAseq, RSEM batch normalized, in patients from the TCGA breast cancer dataset, categorized by breast cancer subtype. Again, the Student *t*-test *p*-value significance between basal and other TNBC subtypes are displayed in brackets above the subtypes. (**C**) The XPO1 protein expression following 24 h treatment with 200 nM selinexor or DMSO control for 4 TNBC cell lines: BT-549, HCC-1806, CAL-120 and MDA-MB-468. Protein expression was quantified from 3 biological replicates per cell line and is displayed in the bar graph to the right. (**D**) *XPO1* mRNA level gene expression and leptomycin B sensitivity (presented as the area under the drug treatment response curve (AUC) correlation in a collection of CCLE breast cancer cell lines. (**E**) The XPO1 protein level expression and leptomycin B sensitivity (presented as the area under the drug treatment response curve (AUC) correlation in a collection of CCLE breast cancer cell lines.

Following the validation of selinexor sensitivity in TNBC cell lines, we next sought to explore the mechanism behind TNBC sensitivity to XPO1 inhibition. We first examined the drug target, namely the XPO1 gene expression levels by breast cancer subtype across the patient samples in METABRIC and TCGA (Figure 3A,B). Similar patterns of XPO1 mRNA expression were observed in both clinical studies with the basal-like breast cancer subtype having the highest and normal-like subtype having the lowest expression levels. Student *t*-test results indicate that the expression levels of XPO1 were statistically significant in basal-like breast cancer compared to any other breast cancer subtype or normal tissue.

We also assessed the effect of selinexor on XPO1 at the protein level. We treated TNBC cell lines: BT-549, HCC-1806, CAL-120 and MDA-MB-468, with 200 nM selinexor or DMSO control, collected whole-cell protein lysate and immunoblotted for XPO1 protein expression (Figure 3C). All 4 TNBC cell lines expressed XPO1 and the drug treatment decreased XPO1 protein abundance. This is consistent with previous in vitro studies which showed that XPO1 is degraded after selinexor treatment in human glioblastoma cell lines [28]. 

Given these results, we investigated the degree to which TNBC sensitivity to XPO1 inhibitors can be explained by the XPO1 expression alone. Using publicly available XPO1 expression data and leptomycin B sensitivity information in a collection of breast cancer cell lines from the CCLE and CTRPv2, we performed a correlation analysis between XPO1 expression (at mRNA and protein level) and measured the cellular sensitivity to leptomycin B (Figure 3D,E). Interestingly, neither mRNA nor protein levels of XPO1 expression correlated with the leptomycin B sensitivity, suggesting that the XPO1 expression alone does not explain TNBC sensitivity to XPO1 inhibition.

### 3.4. RNA-Sequencing Across Four TNBC Cell Lines Reveals Significant Changes to NF-kB Signaling Following Selinexor Treatment

**Figure 4:**
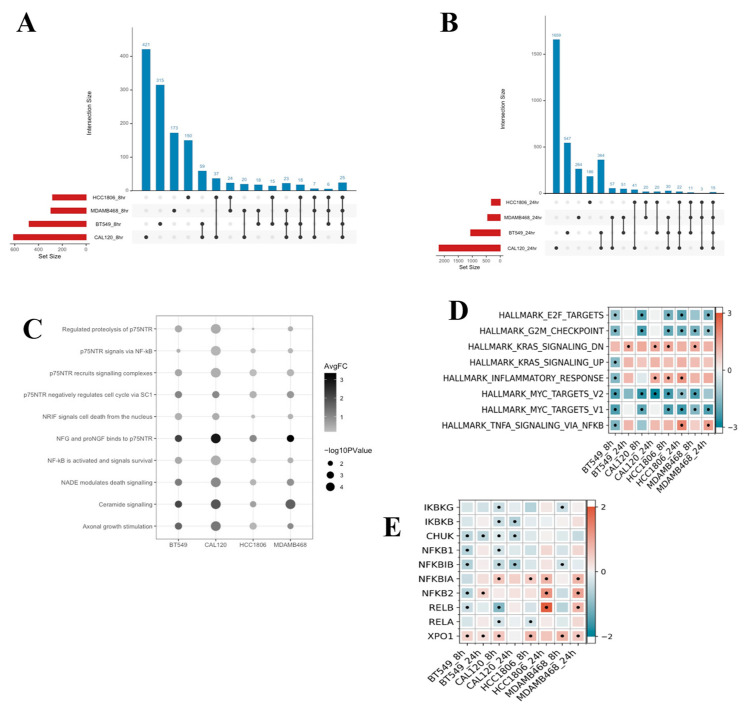
RNA-sequencing across 4 cell lines reveals significant changes to NF-kB signaling following selinexor treatment. (**A**) Upset plot representing significant differentially expressed genes across each cell line at the 8 h time point. Genes identified as significantly differentially expressed were filtered to genes with an absolute log2 fold change > 1, and an FDR *p*-adj < 0.05. Number of significantly differentially expressed genes per cell line is noted on the far left as set size. Interaction size indicates the number of genes identified to be present in the corresponding cell lines indicated below each bar. (**B**) The upset plot of significant differentially expressed genes across each cell line at the 24 h time point filtered in the same way as the 8 h time point. (**C**) Dotplot of all significant Reactome pathways in common across all 4 cell lines at a *p*-value < 0.05. Color gradient of each dot denotes the average fold change whereas dot size represents the *p*-value significance. (**D**) All significant GSEA pathways among 4 cell lines across 2 time points using the MSigDB Hallmark gene sets. Coloration indicates pathway enrichment with positive enrichment shown in red and negative enrichment in blue. A black dot signifies a significant value of a *p*-adj < 0.05 (**E**) Summary of DESeq2 differentially expressed selinexor-treated compared to DMSO control NF-kB pathway genes among 4 cell lines across 2 time points. Coloration indicates upregulation (red) and downregulation (blue) of each gene. A black dot signifies gene significance at an FDR *p*-adj < 0.05.

To explore the mechanism of action of XPO1 inhibitor sensitivity in TNBC, we examined mRNA expression perturbation by selinexor treatment in four TNBC cell lines (BT-549, CAL-120, HCC-1806 and MDA-MB-468) at 8 and 24 h after treatment. The TNBC cell lines were chosen to capture the variability in TNBC. Differential gene expression analysis was performed between selinexor-treated and DMSO control samples for each cell line using DESeq2. Time as a covariate was also assessed (Supplemental Figure S2). To compare and summarize all significantly differentially expressed genes for each cell line, we filtered genes by an FDR-adjusted *p*-value of less than 0.05 and an absolute log2fold change greater than 1. The results are visualized in the upset plots (Figure 4A,B). In total, there were 3934 significantly differentially expressed unique genes among the 4 cell lines and across the 2 time points. We identified nine genes whose expression level significantly changed in all four cell lines and time points (Supplemental Figure S3). Among them, one of the most significant findings was centered around the *NGFR* gene, which showed a very significant adjusted *p*-value and a large effect size (BT-549: *p*-adj = 7.62 × 10^−195^, log2FC = 6.53, CAL-120: *p*-adj = 8.65 × 10^−47^, log2FC = 6.18, HCC-1806: *p*-adj = 5.59 × 10^−22^, log2FC = 2.70, MDA-MB-468: *p*-adj = 2.89 × 10^−16^, log2FC = 3.45). 

In addition to looking at the differentially expressed genes using DESeq2, we also performed a gene set analysis using ReactomeGSA version 1.18.0 for all cell lines and accounting for time points. ReactomeGSA resulted in the following number of significant pathways per cell line: 69 for BT-549, 74 for CAL-120, 75 for HCC-1806 and 62 for MDA-MB-468. To identify the most robust pathway changes due to selinexor perturbation, we focused on the pathways significant across all cell lines. From this, we identified 10, which are visualized in Figure 4C. All 10 pathways were upregulated across each cell line. Interestingly, 7/10 upregulated pathways directly surround p75NTR, another name for NGFR which suggests consistency with our DESseq2 result. “NFG and proNGF binds to p75NTR” upregulation, which was the most changed in terms of the average fold change and *p*-value significance signifying activation of NGFR. Among the 10 common gene sets, the upregulation of “P75NTR signals via NF-kB” and “NF-kB is activated and signals survival” indicates changing NF-kB pathway activity in relationship to the changing NGFR. Indeed, interaction between NGFR and the NF-kB pathway has been previously established [13,15,17,18]. 

We also performed a gene set enrichment analysis (GSEA) for all cell lines and time points using the MSigDB hallmark gene sets for analysis. GSEA resulted in eight significant gene sets (Figure 4D). Comparing pattern of change between 8 h and 24 h for each cell line, similar pathway enrichment was noted among them. “TNF via NF-kB signaling” reported to be significant in three of the four cell lines and the enrichment became more positive between the time points. Since NGFR is a member of the TNF superfamily, this is consistent with our previously described DESeq2 and ReactomeGSA results. Moreover, we observed a positive enrichment score for “KRAS signaling down” in three of the four cell lines and a negative enrichment score for “KRAS signaling up” in the last remaining cell line. Both indicate a reduction in oncogenic KRAS signaling following selinexor treatment and could be caused by NGFR. Furthermore, we observed significant changes to downstream effectors of the NF-kB pathway related to cell proliferation, survival and inflammation such as E2F targets, G2M checkpoint, Myc targets V1 and V2, and inflammatory response in many of the cell lines and time points. Negative enrichment scores were noted in E2F targets, G2M and Myc targets V1 and V2. Literature states that, in breast cancer, the overactivation of NF-kB leads to the activation of these pathways [29]. Therefore, the inhibition of NF-kB activity could be a possible explanation for the decreases seen in the downstream effectors.

To further evaluate the connection among XPO1 inhibitor sensitivity, *NGFR* and NF-KB pathway, we found that while *NGFR* itself does not have an NES, which means that XPO1 and NGFR have no direct interaction, a major regulator of the NF-kB pathway, NFKBIA, possesses one. This, combined with the fact that we identified evidence of significant changes to the NF-kB pathway activity and downstream effectors from both ReactomeGSA and GSEA, persuaded us to examine a collection of canonical and non-canonical NF-kB pathway genes to see whether they are differentially expressed after selinexor treatment (Figure 4E). Overall, we found that the major NF-kB genes expression levels are changing after the selinexor treatment and that there are consistencies in the patterns of change across time and cell line models. Additionally, each gene is significantly differentially expressed in at least one cell line, suggesting that the NF-kB pathway is being altered. With regard to the canonical NF-kB pathway, we identified that the *NFKBIA* gene expression was significantly upregulated in three of the four cell lines and across all cell lines, and *NFKBIA* became more upregulated over time. Meantime, we did not observe significant changes for the primary binding partner of *NFKBIA*, RELA, in most samples. Assessment of the other NF-kB nuclear translocation regulator *IKBKG*, encoding for *NEMO*, only showed significant downregulation in two of the eight samples. In the assessment of the non-canonical NF-kB pathway genes, *NFKB2* also appeared to become upregulated across time points and was significant in three of the four cell lines. Its binding partner *RELB*, unlike *RELA*, was significantly differentially expressed across all four cell lines and became more upregulated across time points. For the expression of *CHUK*, encoding for *IKKa*, the regulator of non-canonical NF-kB translocation, we observed significant downregulation in two of the four cell lines. In summary, many of the upregulated genes are those which decrease NF-kB pathway activity and the downregulated genes are those which activate NF-kB pathway activity. This suggests that selinexor treatment may decrease NF-kB pathway activity.

Taking together, we hypothesize that TNBC selinexor sensitivity is at least in part due to the inhibition of NF-kB activity via the inhibition of the nuclear export of NFKBIA, enabling it to bind to nuclear NF-kB and inhibiting its gene transcription and the blockage of the phosphorylation of NFKBIA, protecting it from degradation.

### 3.5. Selinexor Treatment Increased NFKBIA Nuclear Retention in TNBC Cell Lines

**Figure 5:**
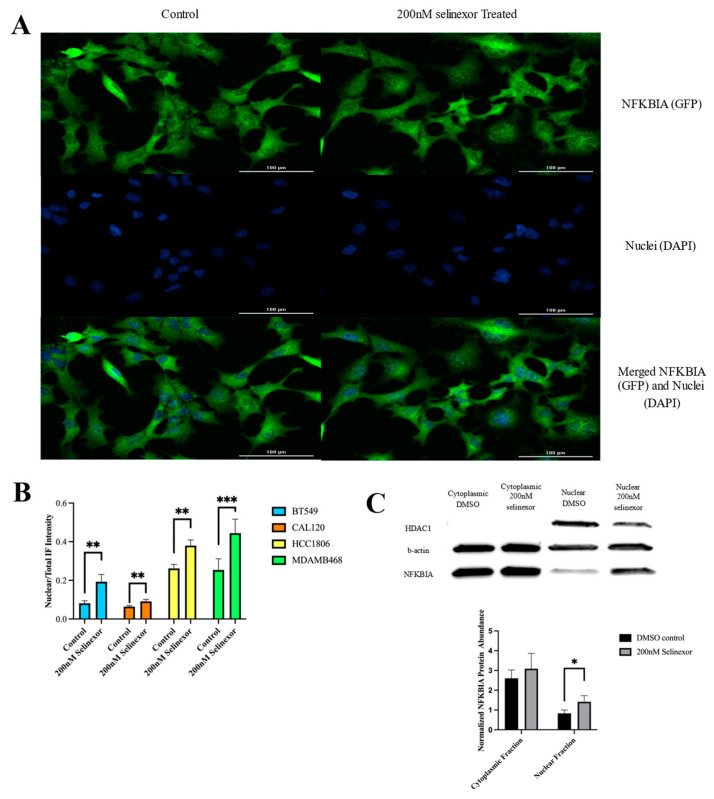
Immunohistochemistry staining and the Western blot of NFKBIA shows nuclear retention following selinexor treatment in 4 TNBC cell lines. (**A**) Immunofluorescence (IF) of BT-549 cell line using BioSpa Cytation shows increased NFKBIA (GFP-labeled) within the nucleus following selinexor treatment. Columns represent the treatment condition and horizontal images were taken from the same section and overlayed. IF images of CAL-120, HCC-1806 and MDA-MB-468 can be found in Supplemental Figure S4. Nuclear staining was obtained using Hoechst by the DAPI channel. (**B**) The quantification of the IF signal across the four cell lines for NFKBIA abundance within the nucleus. Nuclear to total localization was identified using the overlap of the GFP signal and Hoechst nuclei staining and then normalized to the total GFP signal per cell. All four cell lines displayed significantly higher levels of nuclear NFKBIA compared to the control (Student *t*-test, *p*-values: <0.01). (**C**) Western blot of cytoplasmic and nuclear protein fractionization of BT-549 post-24 h treatment with 200 nM selinexor or DMSO control. B-actin was used as a cytoplasmic loading control while HDAC1 was used as a nuclear control. The NFKBIA signal was normalized to loading control and *p*-value significance across 3 biological replicates was significant by Student *t*-test at 0.046. Associated normalized protein abundance is also represented in the bar graph directly below the Western blot. An additional Western blot of HCC-1806, analyzed with the same protocol stated previously, can be found in Supplemental Figure S4. The number of * in each bar graph indicate *p*-value significance with * indicating <0.05, ** <0.01 and *** <0.001.

Since XPO1 functions by exporting cargo proteins outside of the nucleus, we tested the sub-cellular localization of NFKBIA after treatment with selinexor. Following 24 h drug treatment, we found significant increases in nuclear levels of NFKBIA in all four cell lines (immunofluorescence staining, Figure 5A,B, Supplemental Figure S4). Additionally, to validate the immunofluorescence results, we conducted Western blotting on nuclear and cytoplasmic fractioned protein and found nuclear retention for NFKBIA in both BT-549 and HCC-1806 cell lines. Note that, although the same nuclear retention trend was observed, the results were only significant in BT-549 cells (Figure 5C *p*-value < 0.05). The examination of the cytoplasmic fractions for each cell line did not differ, indicating that only nuclear amounts of NFKBIA are increasing (Figure 5C, Supplemental Figure S4).

### 3.6. shRNA Knockdown of NFKBIA in TNBC Cell Lines Decreases Selinexor’s Drug Efficacy

**Figure 6:**
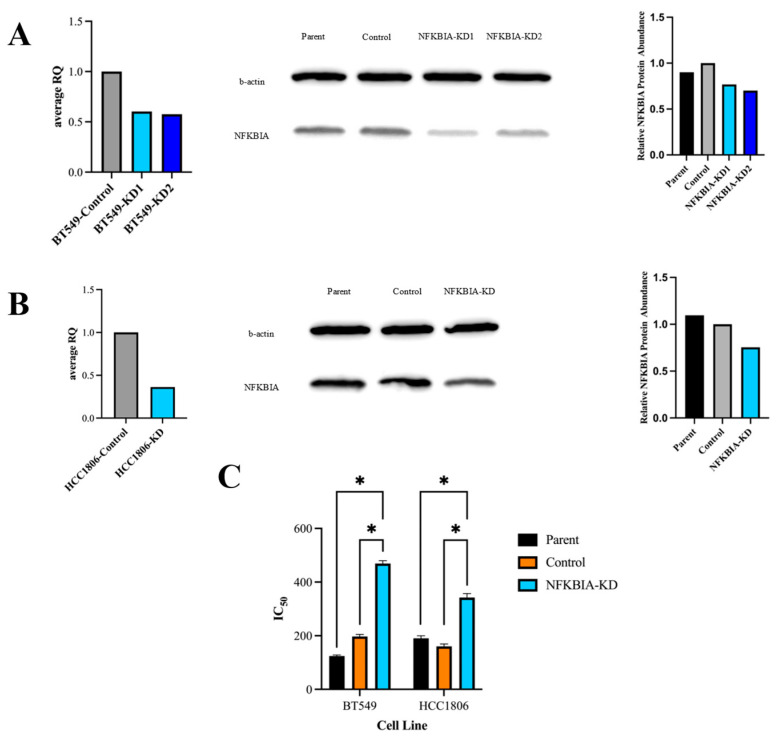
Knockdown of *NFKBIA* decreases sensitivity to selinexor. (**A**) BT-549 NFKBIA gene and protein expression following shRNA knockdown assessed using RT-qPCR (left panel) and Western blot (middle and right panels). Samples of the control scrambled insert as well as NFKBIA knockdown were collected and analyzed at both the gene and protein levels; both confirmed the decreased levels of NFKBIA within the generated knockdown cell line compared to the control scrambled. (**B**) Similarly to (**A**), the NFKBIA gene and protein expression following shRNA knockdown in HCC-1806 cells were assessed via RT-qPCR and Western blot. The knockdown-generated version of the cell line showed decreased levels of NFKBIA at both the gene and protein levels. (**C**) IC50 results for parent, control generated and NFKBIA knockdown-generated cells in both BT-549 and HCC-1806 are shown with standard error bars and factoring in biological replicates. Like Figure 2, 10-point dose–response curves were fit testing the viability of all cell lines after 72 h treatment of between 0 and 3200 nM selinexor. In both cell lines, NFKBIA knockdown displayed statistically significant higher IC50 values compared to either parent or control as indicated by * in (**C**) (*p*-value: < 0.0001).

To further test whether NFKBIA is necessary for selinexor response in TNBC cells, we performed gene knock-down experiments in BT-549 and HCC-1806 cells. In both cell lines, the NFKBIA knockdown did not significantly change the cell growth rate compared to control insert scrambled cells (Supplemental Figure S5). NFKBIA knockdown efficiency was confirmed by RT-qPCR and Western blot. Specifically, RT-qPCR confirmed a knockdown efficacy of 45% and 64% in BT-549 and HCC-1806, respectively, at the mRNA level (Figure 6A,B). Western blots indicated a 20% and a 31% knockdown in BT-549 and HCC-1806, respectively (Figure 6A,B). To examine whether decreased levels of NFKBIA expression renders TNBC cells resistant to XPO1 inhibition, we treated BT-549 and HCC-1806 NFKBIA knockdown cell lines with selinexor and measured the cellular viability. We found that NFKBIA knockdown cells were significantly less sensitive to XPO1-inhibition-mediated cell death in both BT-549 and HCC-1806 with the reported IC50s of knockdown to control being 469 nM to 197 nM (*p*-value < 0.0001) and 342 nM to 160 nM (*p*-value < 0.0001), respectively (Figure 6C). These decreased cellular sensitivities towards selinexor treatment in the presence of NFKBIA knockdown further support the functional role of NFKBIA in TNBC cell line sensitivity to selinexor.

## 4. Discussion

Compared to other breast cancer subtypes, TNBC continues to be the most aggressive, have high metastatic potential, and poorer prognosis [3]. Additionally, with the lack of ER, PR and HER2 receptors, as well as a diversity in molecular subtypes, innovations in treatment for TNBC lags behind those of its ER, PR and HER2 positive counterparts [4]. To date, systemic chemotherapy is still the primary option in neoadjuvant and adjuvant settings. With the availability of high-throughput drug screens combined with the emergence of computational modeling, this has allowed us to take a novel approach in seeking pharmacological candidates for the treatment of TNBC [10,30]. We were able to impute drug sensitivity scores for breast cancer patient tumors in two independent datasets, show consistencies between them and identify drugs with preferential sensitivity towards TNBC tumors. Among the hundreds of drugs screened, XPO1 inhibitors are represented by leptomycin B and appear to be one of the top candidates with the most significant *p*-value and/or effect size. We subsequently investigated selinexor, a XPO1 inhibitor that has already been approved by FDA to treat MM [13]. The results of this paper indicate that selinexor is computationally predicted and validated to be effective in TNBC patients, as experimentally validated in a broad panel of TNBC cell lines. We also identify NF-kB signaling a primary driver of its sensitivity.

Since selinexor’s approval in MM, there has been increasing interest in using XPO1 inhibition in other cancer context [2,15,31,32,33,34]. However, the exploration of XPO1 inhibitors in TNBC has been limited. Similarly to our initial in vitro screening of seven TNBC cell lines across a variety of subtypes, other studies examining selinexor have found that it promoted apoptosis and decreased cell viability and growth in variety of in vitro and in vivo models [34,35,36]. Our work added to the existing literature by exploring the mechanism of action for which selinexor achieves its efficacy in TNBC. By conducting RNA sequencing experiments in multiple cell line models with longitudinal expression assessment, we identified the importance of NGFR, the NF-kB pathway, and specifically NFKBIA in causing selinexor-mediated TNBC cell death. 

Our results indicate that XPO1 expression alone cannot explain TNBC sensitivity to XPO1 inhibition. Previous papers investigating the correlation of XPO1 expression in various cancers with selinexor sensitivity appears to be inconclusive with some reporting a positive correlation and others not finding any [14,37]. Although a separate cancer context, our analysis aligned with the neuroblastoma study by Galinski et al., and did not identify a correlation between XPO1 expression and selinexor sensitivity, at neither the gene nor protein levels. This suggests that TNBC XPO1 inhibitor sensitivity is independent of XPO1 expression and that XPO1 expression alone cannot be a predictive marker of response. Additionally, although XPO1 is often overexpressed in cancers and is responsible for carrying a broad range of proteins and mRNA species, our correlation results suggest that it is the cargo which XPO1 transports that is of importance—rather than XPO1 itself—in determining therapeutic effectiveness. 

Based on our RNA-seq analysis, it is quite evident that the interplay between NGFR and the NF-kB pathway, as all common significant results across model systems, can be explained by these two. While the GSEA displayed a diverse array of major pathways, all are downstream of NF-kB, except for KRAS signaling, which may be attributed to the upregulation in NGFR. As previously reported, competitive binding between TrkA, the upstream receptor leading to KRAS activation, and NGFR, was a key determinant of NGF initiating cellular death or survival [38]. Therefore, increased NGFR but not TrkA could indicate lower levels of NGF binding to the TrkA receptor, thus leading to a reduction in KRAS signaling. Moreover, the overall direction in which pathways and genes were upregulated or downregulated was consistent with a reduction in NF-kB pathway activity; downstream effectors were downregulated and genes which inhibit the NF-kB pathway were upregulated. Specifically, within breast cancer, the NF-kB pathway is reported to promote tumor growth and angiogenesis [39]. Furthermore, it has been stated that TNBC patient populations have higher levels of constitutively active NF-kB signaling compared to other subtypes [29]. This may partially explain selinexor’s preferential sensitivity towards TNBC patient populations. 

While this paper presents multiple instances evidencing NF-kB downregulation, interestingly, a few pathways showed an enrichment of NF-kB activity. “NFkB is activated and signals survival” from the ReactomeGSA and the upregulation of “hallmark inflammatory response” from GSEA. Since it is well known that the NF-kB pathway can cause both cell death and survival in a context-dependent manner, it is plausible that cell death due to selinexor also induces cellular stress and therefore increased cytokine signaling, leading to the two above pathways becoming significant. Further investigation of the differentially expressed genes within these pathways shows evidence to support this.

The attribution of specific target proteins of selinexor sensitivity has been explored in a few different cancer cell types, mainly leukemias, lymphomas and myelomas [14,17,19,40]. Thus far, hypotheses of the mechanism causing TNBC sensitivity to selinexor has been proposed based on the known TNBC molecular characteristics [2,20,41]. Our study took an unbiased approach by examining multiple TNBC models and various drug exposure time points to identify the TNBC constant contributors to selinexor sensitivity. Our findings suggest that the inhibition of the NF-kB pathway via the nuclear retention of NFKBIA is vital in determining TNBC selinexor sensitivity. Evidence of this is that, although our shRNA knockdown of NFKBIA resulted in only a 20% reduced expression in BT-549 and a 31% reduction in HCC-1806 compared to control scrambled, we were able to observe a significant decrease in sensitivity to selinexor in knockdown models. As only a small reduction in NFKBIA expression led to increased selinexor resistance, this supports the hypothesis of how critical NFKBIA is for TNBC selinexor sensitivity. While this is the first study to report this specific target protein in TNBC, NFKBIA has been implicated within the mechanism of selinexor sensitivity for other cancer contexts, such as non-Hodgkin lymphoma, MM and high-grade glioma [17,37,40,42,43]. Previous work has demonstrated that NFKBIA can inhibit NF-kB activity not only within the cytoplasm, but also in the nucleus if it is present [18]. Therefore, the accumulation of NFKBIA within the nucleus due to XPO1 inhibition in TNBC cells may explain the reduction in NF-kB transcriptional activity similar to that seen in other cancer contexts. 

## 5. Conclusions

In conclusion, we utilized high-throughput drug screening data to predict the TNBC patient tumor’s response to hundreds of compounds and identified XPO1 inhibitors as preferentially sensitive in these patients. We validated XPO1 inhibitor response in a heterogeneous collection of TNBC cell lines. We further investigated the mechanism causing TNBC XPO1 inhibitor sensitivity, and identified changes in the NF-kB pathway as well as the interaction between NGFR and NF-kB as critical. Increases in resistance to selinexor following the knockdown of NFKBIA confirms the importance of NFKBIA in the context of TNBC selinexor sensitivity. Results from this study provided additional insights into the therapeutic landscape of TNBC treatment with selinexor.

## Data Availability

All data generated or analyzed during this study are included in the published article. Samples generated for RNA-sequencing are uploaded to GEO and available for download under the accession ID GSE278241.

## Supplementary Materials

The following supporting information can be downloaded at https://www.mdpi.com/article/10.3390/cancers16233980/s1, Figure S1: Breast cancer cell lines reported EC50 for XPO1 inhibitor compounds screened in the CTRP; Figure S2: Volcano plots of differentially expressed genes with time as a covariate; Figure S3: Dot plot of all common genes significantly changed across all time points and cell lines; Figure S4: Localization of NFKBIA changes following selinexor treatment in 3 additional cell lines; Figure S5: shRNA knockdown cells do not differ in cell growth rate compared to their respective control.

## References

[1] Sung H., Ferlay J., Siegel R.L., Laversanne M., Soerjomataram I., Jemal A., Bray F. (2021). Global Cancer Statistics 2020: GLOBOCAN Estimates of Incidence and Mortality Worldwide for 36 Cancers in 185 Countries. CA Cancer J. Clin..

[2] Soung Y.H., Kashyap T., Nguyen T., Yadav G., Chang H., Landesman Y., Chung J. (2017). Selective Inhibitors of Nuclear Export (SINE) compounds block proliferation and migration of triple negative breast cancer cells by restoring expression of ARRDC3. Oncotarget.

[3] Dent R., Trudeau M., Pritchard K.I., Hanna W.M., Kahn H.K., Sawka C.A., Lickley L.A., Rawlinson E., Sun P., Narod S.A. (2007). Triple-Negative Breast Cancer: Clinical Features and Patterns of Recurrence. Clin. Cancer Res..

[4] Obidiro O., Battogtokh G., Akala E.O. (2023). Triple Negative Breast Cancer Treatment Options and Limitations: Future Outlook. Pharmaceutics.

[5] Zagami P., Carey L.A. (2022). Triple negative breast cancer: Pitfalls and progress. npj Breast Cancer.

[6] Orrantia-Borunda E., Anchondo-Nuñez P., Acuña-Aguilar L.E., Gómez-Valles F.O., Ramírez-Valdespino C.A., Mayrovitz H.N. (2022). Subtypes of Breast Cancer. Breast Cancer.

[7] DiMasi J.A., Grabowski H.G., Hansen R.W. (2016). Innovation in the pharmaceutical industry: New estimates of R&D costs. J. Health Economics.

[8] DiMasi J.A., Reichert J.M., Feldman L., Malins A. (2013). Clinical Approval Success Rates for Investigational Cancer Drugs. Clin. Pharmacol. Ther..

[9] Yamaguchi S., Kaneko M., Narukawa M. (2021). Approval success rates of drug candidates based on target, action, modality, application, and their combinations. Clin. Transl. Sci..

[10] Maeser D., Gruener R.F., Huang R.S. (2021). oncoPredict: An R package for predicting in vivo or cancer patient drug response and biomarkers from cell line screening data. Brief. Bioinform..

[11] Gruener R.F., Ling A., Chang Y.F., Morrison G., Geeleher P., Greene G.L., Huang R.S. (2021). Facilitating Drug Discovery in Breast Cancer by Virtually Screening Patients Using In Vitro Drug Response Modeling. Cancers.

[12] Ling A., Huang R.S. (2020). Computationally predicting clinical drug combination efficacy with cancer cell line screens and independent drug action. Nat. Commun..

[13] Azizian N.G., Li Y. (2020). XPO1-dependent nuclear export as a target for cancer therapy. J. Hematol. Oncol..

[14] Zhao L., Luo B., Wang L., Chen W., Jiang M., Zhang N. (2021). Pan-cancer analysis reveals the roles of XPO1 in predicting prognosis and tumorigenesis. Transl. Cancer Res..

[15] Landes J.R., Moore S.A., Bartley B.R., Doan H.Q., Rady P.L., Tyring S.K. (2023). The efficacy of selinexor (KPT-330), an XPO1 inhibitor, on non-hematologic cancers: A comprehensive review. J. Cancer Res. Clin. Oncol..

[16] Sun Q., Chen X., Zhou Q., Burstein E., Yang S., Jia D. (2016). Inhibiting cancer cell hallmark features through nuclear export inhibition. Signal Transduct. Target. Ther..

[17] Wang A.Y., Liu H. (2019). The past, present, and future of CRM1/XPO1 inhibitors. Stem Cell Investig..

[18] Kashyap T., Argueta C., Aboukameel A., Unger T.J., Klebanov B., Mohammad R.M., Landesman Y. (2016). Selinexor, a Selective Inhibitor of Nuclear Export (SINE) compound, acts through NF-κB deactivation and combines with proteasome inhibitors to synergistically induce tumor cell death. Oncotarget.

[19] Sendino M., Omaetxebarria M.J., Rodríguez J.A. (2018). Hitting a moving target: Inhibition of the nuclear export receptor XPO1/CRM1 as a therapeutic approach in cancer. Cancer Drug Resist..

[20] Cheng Y., Holloway M.P., Nguyen K., McCauley D., Landesman Y., Kauffman M.G., Shacham S., Altura R.A. (2014). XPO1 (CRM1) inhibition represses STAT3 activation to drive a survivin-dependent oncogenic switch in triple negative breast cancer. Mol. Cancer Ther..

[21] Curtis C., Shah S.P., Chin S.F., Turashvili G., Rueda O.M., Dunning M.J., Speed D., Lynch A.G., Samarajiwa S., Yuan Y. (2012). The genomic and transcriptomic architecture of 2,000 breast tumours reveals novel subgroups. Nature.

[22] Weinstein J.N., Collisson E.A., Mills G.B., Shaw K.M., Ozenberger B.A., Ellrott K., Shmulevich I., Sander C., Stuart J.M. (2013). The Cancer Genome Atlas Pan-Cancer Analysis Project. Nat Genet..

[23] cBioPortal for Cancer Genomics. https://www.cbioportal.org/study/summary?id=brca_metabric.

[24] Seashore-Ludlow B., Rees M.G., Cheah J.H., Cokol M., Price E.V., Coletti M.E., Jones V., Bodycombe N.E., Soule C.K., Gould J. (2015). Harnessing Connectivity in a Large-Scale Small-Molecule Sensitivity Dataset. Cancer Discov..

[25] DepMap Data Downloads. https://depmap.org/portal/download/all/.

[26] Johnson W.E., Li C., Rabinovic A. (2007). Adjusting batch effects in microarray expression data using empirical Bayes methods. Biostatistics.

[27] Selinexor. https://go.drugbank.com/drugs/DB11942.

[28] Zhu Z.C., Liu J.W., Yang C., Zhao M., Xiong Z.Q. (2019). XPO1 inhibitor KPT-330 synergizes with Bcl-xL inhibitor to induce cancer cell apoptosis by perturbing rRNA processing and Mcl-1 protein synthesis. Cell Death Dis..

[29] Wang W., Nag S.A., Zhang R. (2015). Targeting the NFκB Signaling Pathways for Breast Cancer Prevention and Therapy. Curr. Med. Chem..

[30] Carnero A. (2006). High throughput screening in drug discovery. Clin. Transl. Oncol..

[31] Garzon R., Savona M., Baz R., Andreeff M., Gabrail N., Gutierrez M., Savoie L., Mau-Sorensen P.M., Wagner-Johnston N., Yee K. (2017). A phase 1 clinical trial of single-agent selinexor in acute myeloid leukemia. Blood.

[32] Aggarwal V., Tang T., Daugaard G., Liu S.V., Ahn J., Besse B., Girard N., Giaccone G., Kim C., Petersen P. (2024). A phase II clinical trial of selinexor in patients with advanced thymoma and thymic carcinoma. J. Clin. Oncol..

[33] (2017). Clinical Trials Using Selinexor-NCI. https://www.cancer.gov/research/participate/clinical-trials/intervention/selinexor.

[34] Shafique M., Ismail-Khan R., Extermann M., Sullivan D., Goodridge D., Boulware D., Hogue D., Soliman H., Khong H., Han H.S. (2019). A Phase II Trial of Selinexor (KPT-330) for Metastatic Triple-Negative Breast Cancer. Oncol..

[35] Arango N.P., Yuca E., Zhao M., Evans K.W., Scott S., Kim C., Meric-Bernstam F. (2017). Selinexor (KPT-330) demonstrates anti-tumor efficacy in preclinical models of triple-negative breast cancer. Breast Cancer Res..

[36] Marijon H., Gery S., Chang H., Landesman Y., Shacham S., Lee D.H., Koeffler H.P. (2021). Selinexor, a selective inhibitor of nuclear export, enhances the anti-tumor activity of olaparib in triple negative breast cancer regardless of BRCA1 mutation status. Oncotarget.

[37] Galinski B., Luxemburg M., Landesman Y., Pawel B., Johnson K.J., Master S.R., Freeman K.W., Loeb D.M., Hébert J.M., Weiser D.A. (2021). XPO1 inhibition with selinexor synergizes with proteasome inhibition in neuroblastoma by targeting nuclear export of IkB. Transl. Oncol..

[38] Yoon S.O., Casaccia-Bonnefil P., Carter B., Chao M.V. (1998). Competitive Signaling Between TrkA and p75 Nerve Growth Factor Receptors Determines Cell Survival. J. Neurosci..

[39] Guo Q., Jin Y., Chen X., Ye X., Shen X., Lin M., Zhang J. (2024). NF-κB in biology and targeted therapy: New insights and translational implications. Sig. Transduct. Target. Ther..

[40] Gandhi U.H., Senapedis W., Baloglu E., Unger T.J., Chari A., Vogl D., Cornell R.F. (2018). Clinical Implications of Targeting XPO1-mediated Nuclear Export in Multiple Myeloma. Clin. Lymphoma Myeloma Leuk..

[41] Martini S., Zuco V., Tortoreto M., Percio S., Campi E., El Bezawy R., Doldi V., Landesman Y., Pennati M., Zaffaroni N. (2021). miR-34a-Mediated Survivin Inhibition Improves the Antitumor Activity of Selinexor in Triple-Negative Breast Cancer. Pharmaceuticals.

[42] DeSisto J.A., Flannery P., Lemma R., Pathak A., Mestnik S., Philips N., Green A.L. (2020). Exportin 1 inhibition induces nerve growth factor receptor expression to inhibit the NF-κB pathway in preclinical models of pediatric high-grade glioma. Mol. Cancer Ther..

[43] Han X., Wang J., Shen Y., Zhang N., Wang S., Yao J., Shi Y. (2015). CRM1 as a new therapeutic target for non-Hodgkin lymphoma. Leuk. Res..

