# Supplementary material for: Discovery, Validation and Mechanistic Study of XPO1 Inhibition in the Treatment of Triple-Negative Breast Cancer
Source: Cancers (Basel). 2024 Nov 27;16(23):3980. doi: 10.3390/cancers16233980 (PMC11640544; doi:10.3390/cancers16233980)
Supplement: Supplementary file 1 [file cancers-16-03980-s001.zip › supplemental figures with legends.pdf]

SUPPLEMENTAL FIGURES/FIGURE LEGENDS

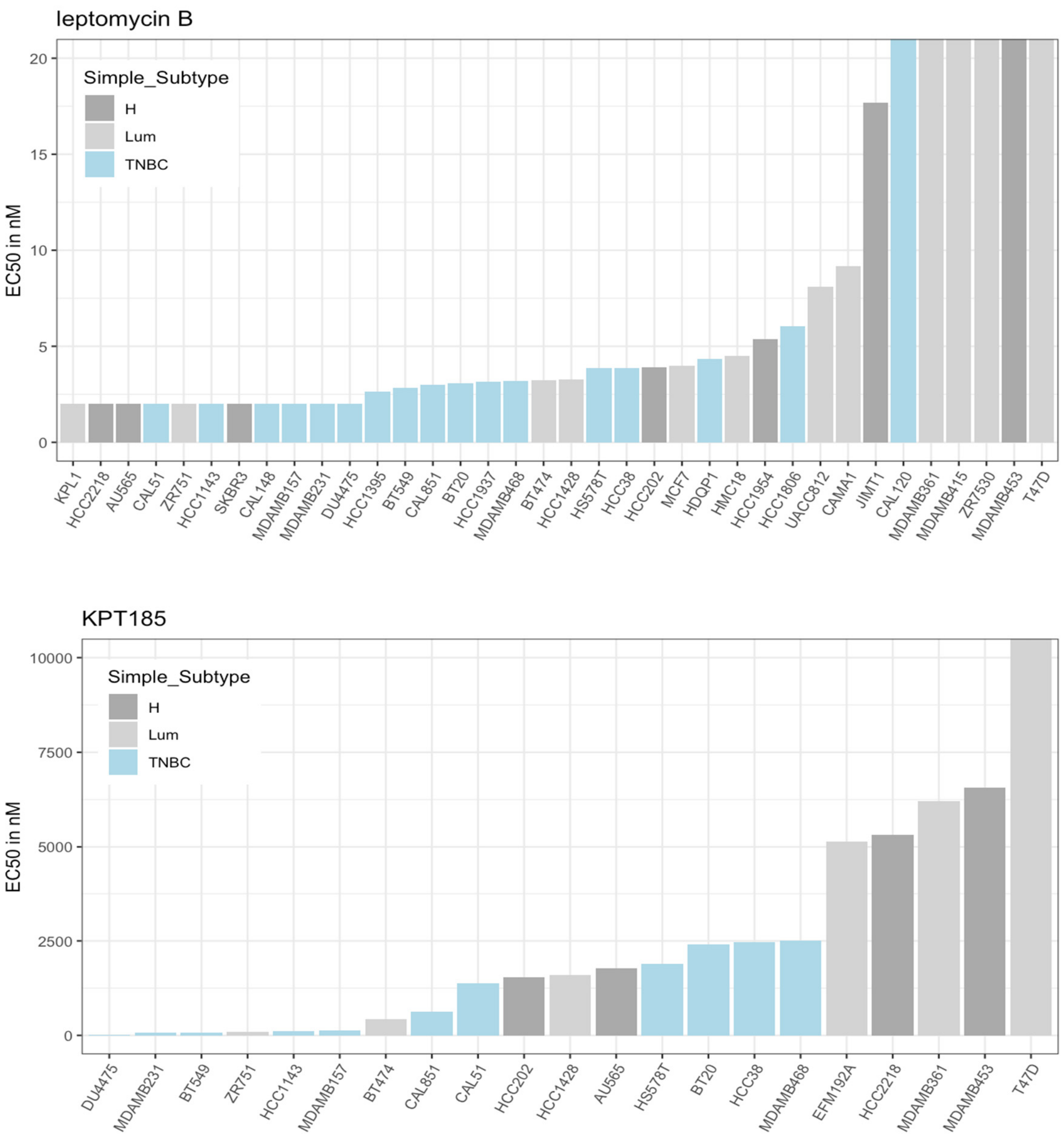

Supplemental Figure S1. Breast cancer cell lines reported EC_{50} for XPO1 inhibitor compounds screened in the CTRP. Reported EC_{50} for XPO1 inhibitors leptomycin B and KPT-185 in all breast cancer cell lines screened within the CTRP. Cell lines are displayed along the x-axis and subtype is denoted by coloration with TNBC represented in blue. Cell lines which continue off the y-axis are greater than log10 fold higher.

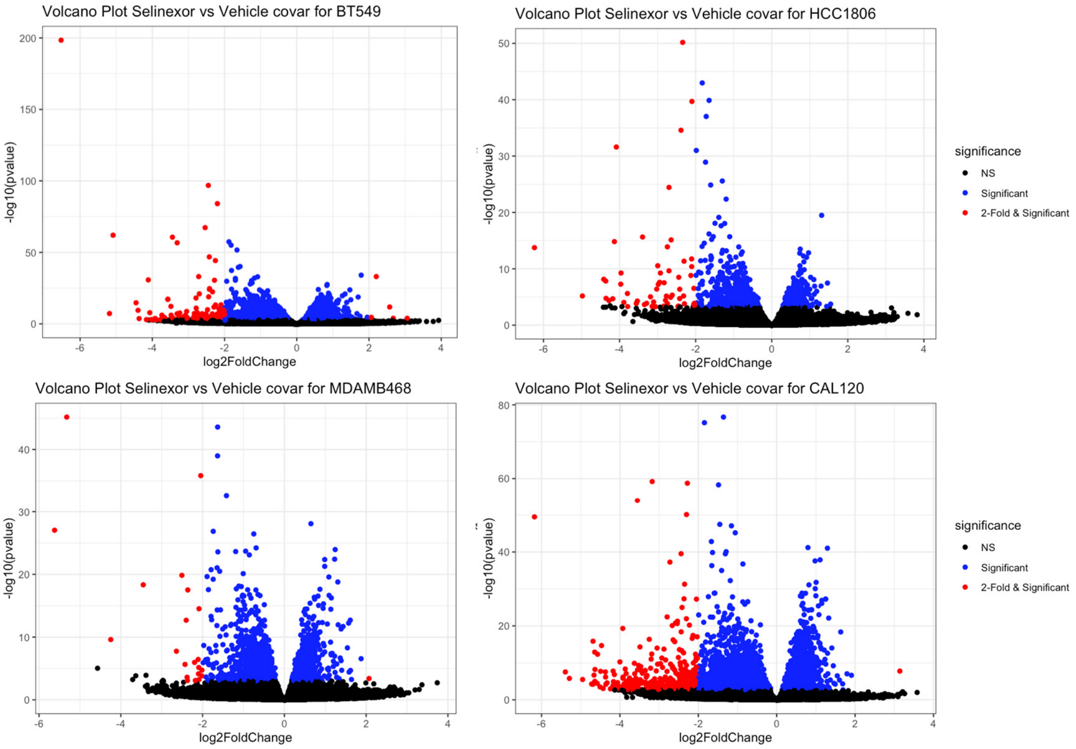

Supplemental Figure S2. Volcano Plots of Differentially Expressed Genes with Time as a Covariate. Differential gene expression analysis between selinexor and DMSO control treated samples was performed on BT-549, HCC-1806, MDA-MB-468, and CAL-120 using DESeq2. For each cell line indicated, the log2fold change (x-axis) is plotted against the log10 of the adjusted p-value (y-axis). Blue indicates genes that achieve a significance of less than 0.10 adjusted p-value and red points are both significant and have an absolute log2 fold change greater than 2 (i.e. a 4-fold change). Note the x- and y-axes are free for each graph.

SUPPLEMENTAL FIGURE S3.

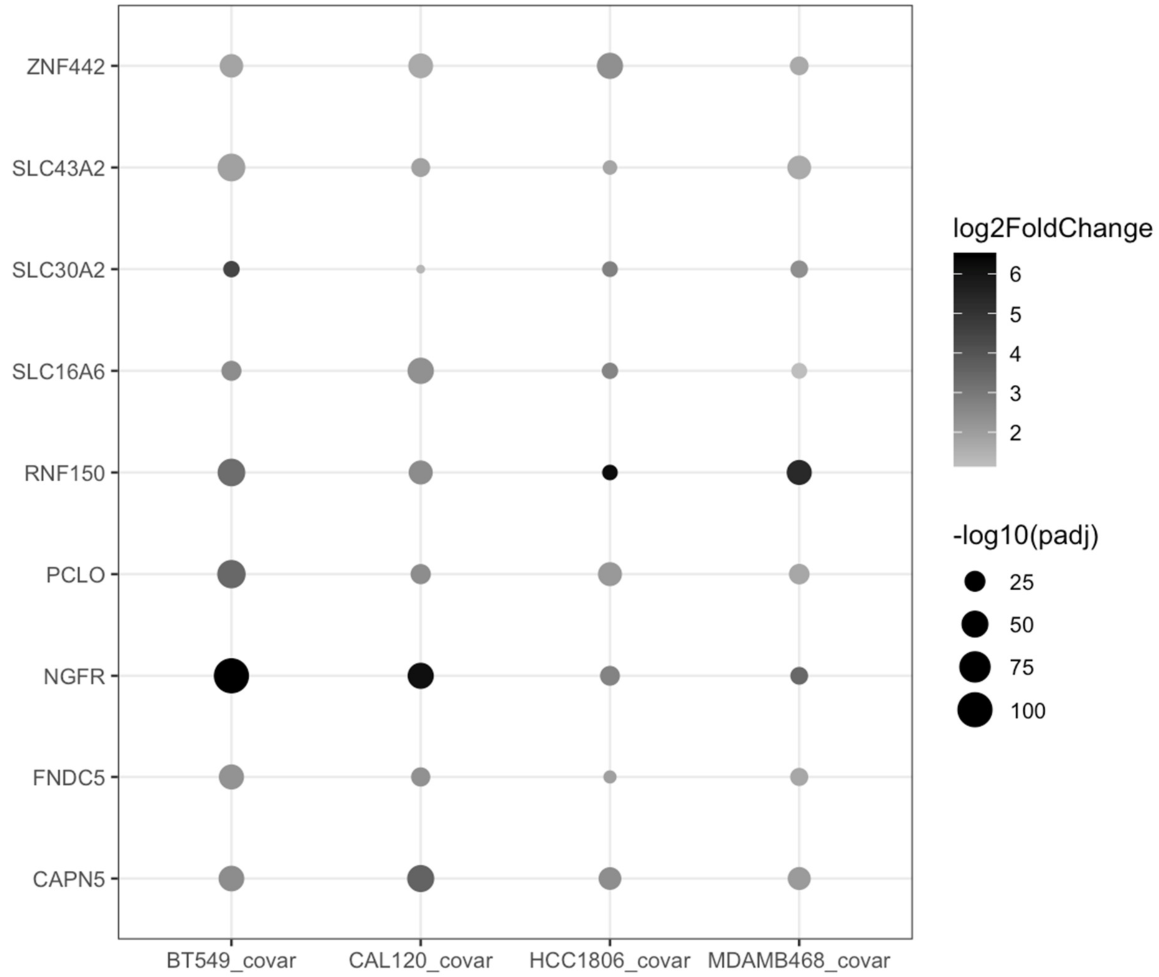

Supplemental Figure S3. Dot plot of all common genes significantly changed across all time points and cell lines. Analysis of the differentially expressed genes filtered down to those commonly significant across both time points and all cell lines. Significance was defined as an adjusted p-value greater than 0.05 and an absolute log2 fold change greater than 1. Displayed above are the 4 cell lines using time as a covariate (x-axis) with a total of 9 genes reported in common (y-axis). The size of each dot indicates the p-value significance (larger is more significant) and color indicating the degree of log2 fold change (darker indicating greater fold change).

SUPPLEMENTAL FIGURE S4.

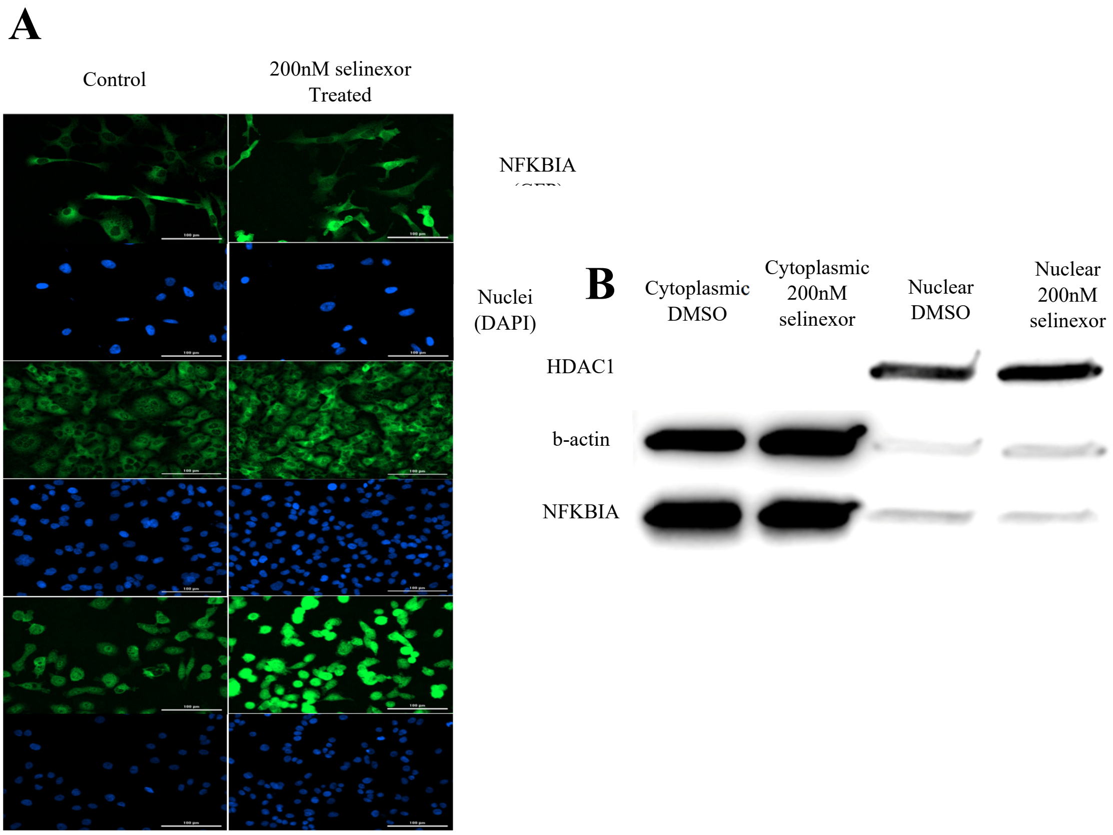

Supplemental Figure S4. Localization of NFKBIA changes following selinexor treatment in 3 additional cell lines. (A) Immunofluorescence of CAL120, HCC1806, and MDAMB468 cell lines respectively using BioSpa Cytation shows increased NFKBIA (GFP-labeled) within the nucleus following selinexor treatment. Nuclear staining was obtained using Hoechst by the DAPI channel. (B) Additional western Blot of cytoplasmic and nuclear protein fractionization of HCC1806 post-24hr treatment with 200nM selinexor or DMSO control. B-actin was used as a cytoplasmic loading control while HDAC1 was used as a nuclear control. NFKBIA signal was normalized to loading control and P-value significance across 3 biological replicates was reported to be 0.1.

SUPPLEMENTAL FIGURE S5.

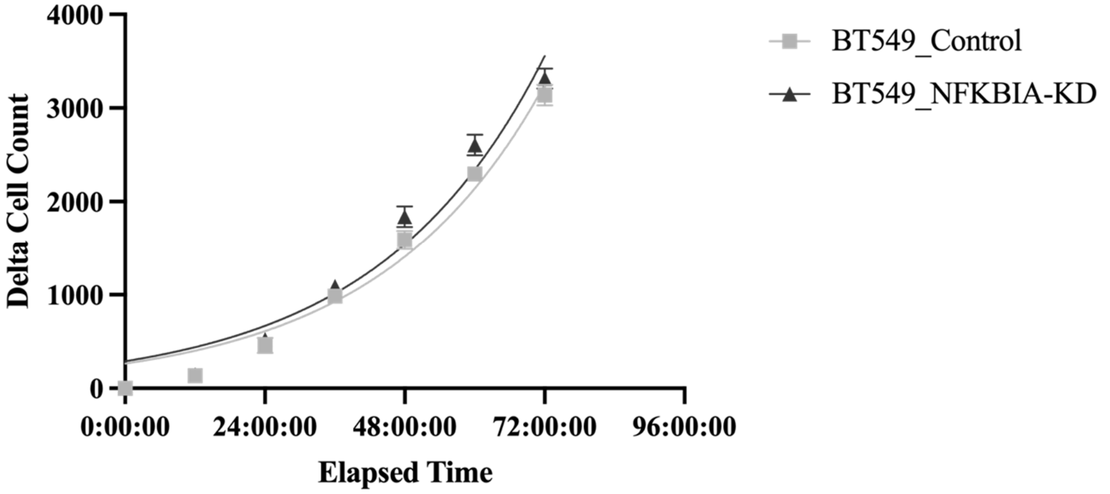

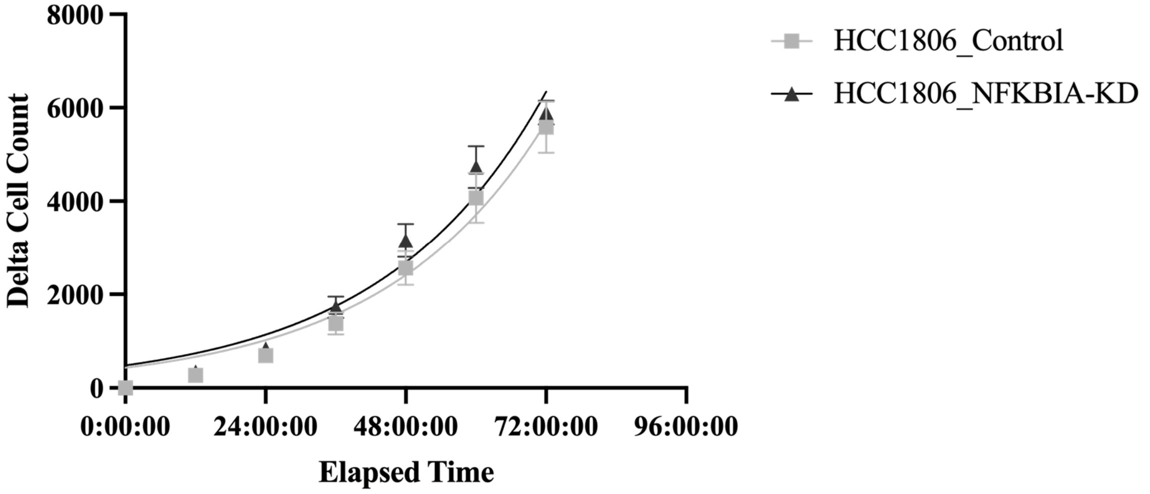

Supplemental Figure S5. shRNA knockdown cells do not differ in cell growth rate compared to their respective control. Growth rates of BT-549 and HCC-1806 NFKBIA knockdown and control cell lines extrapolated using delta cell count across time. Delta cell count defined as number of cells per well divided by number of cells per well at time point 0 to account for slight seeding density variation from well to well. In both cell lines, knockdown did not significantly affect the rate of proliferation compared to control.
