# Supplementary figures and images for: Discovery, Validation and Mechanistic Study of XPO1 Inhibition in the Treatment of Triple-Negative Breast Cancer
Source: Cancers (Basel). 2024 Nov 27;16(23):3980. doi: 10.3390/cancers16233980 (PMC11640544; doi:10.3390/cancers16233980)

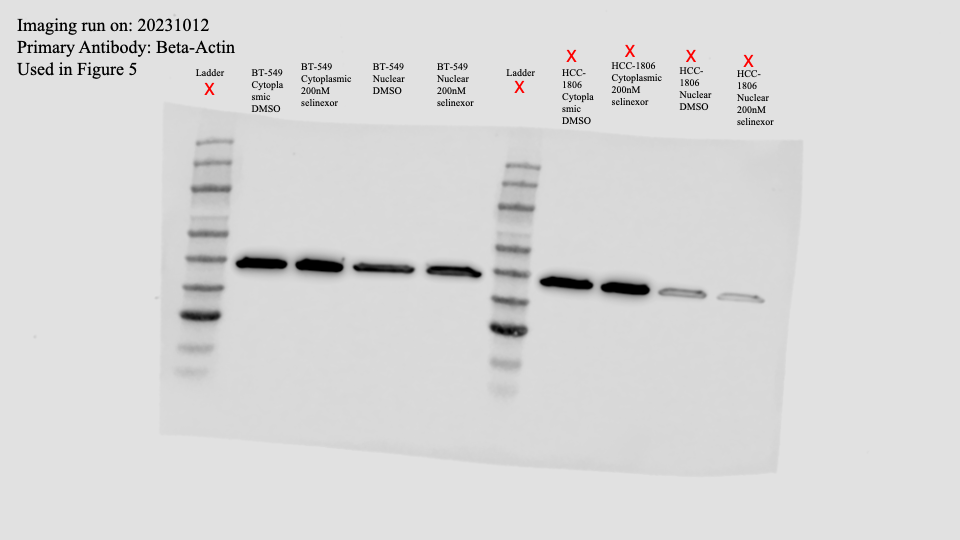

Supplement: Supplementary file 1 [file cancers-16-03980-s001.zip › westerns/DTBActin-B.tiff]

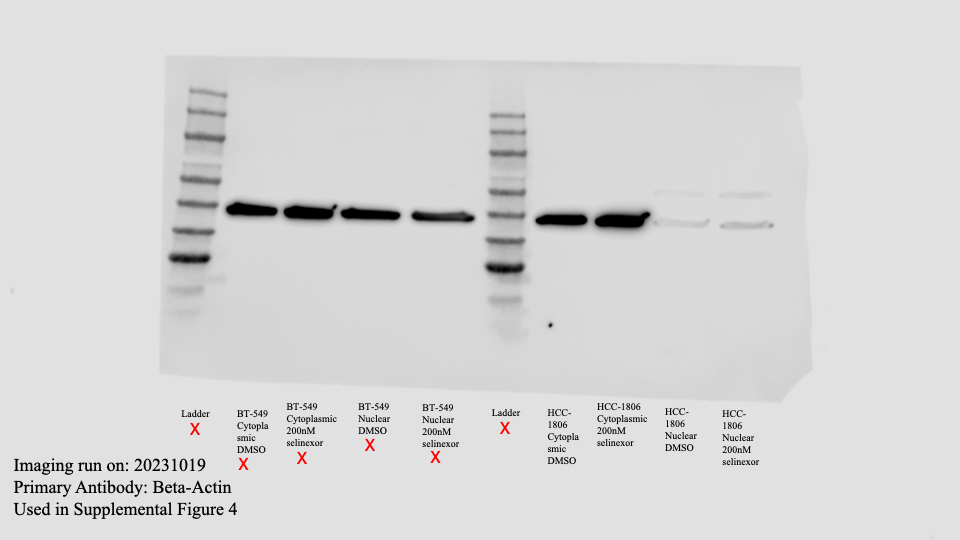

Supplement: Supplementary file 1 [file cancers-16-03980-s001.zip › westerns/DTBActin-H.tiff]

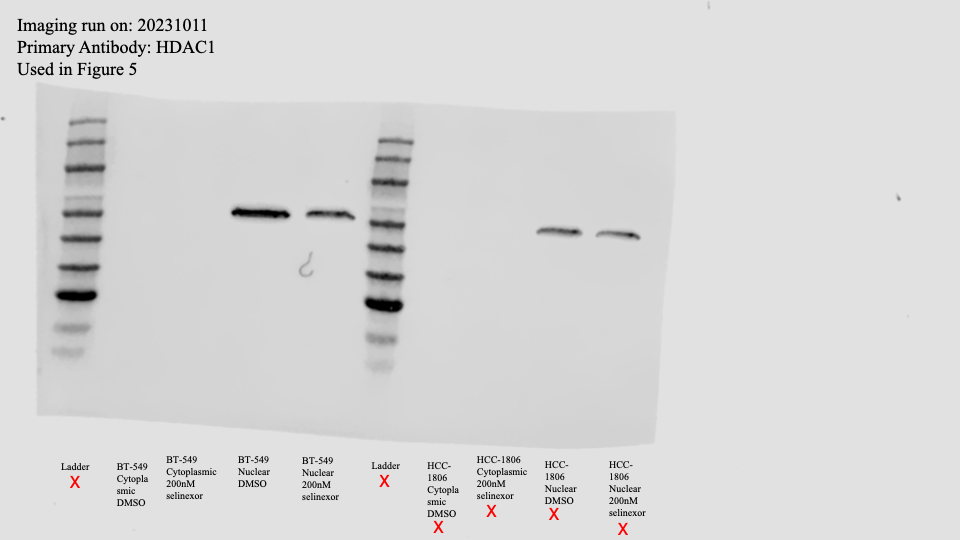

Supplement: Supplementary file 1 [file cancers-16-03980-s001.zip › westerns/DTHDAC1-B.tiff]

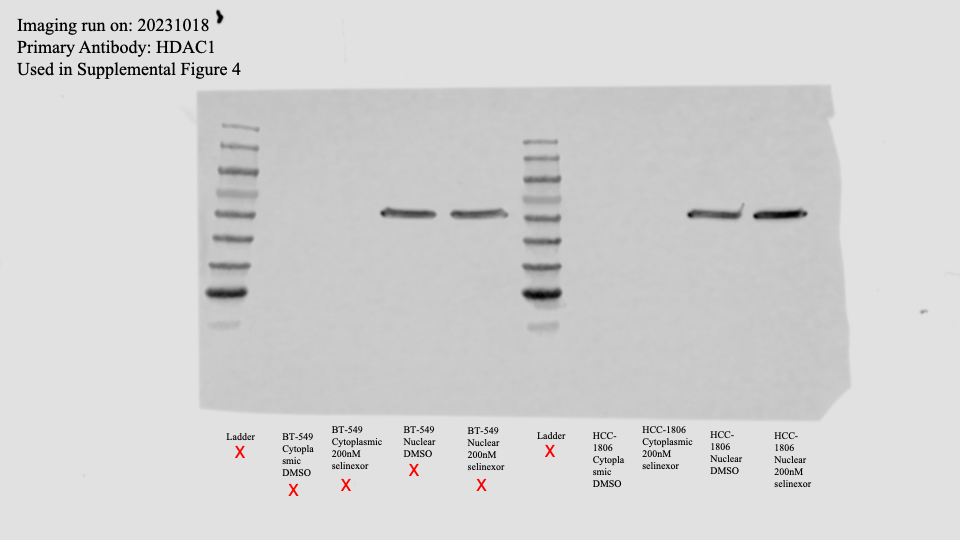

Supplement: Supplementary file 1 [file cancers-16-03980-s001.zip › westerns/DTHDAC1-H.tiff]

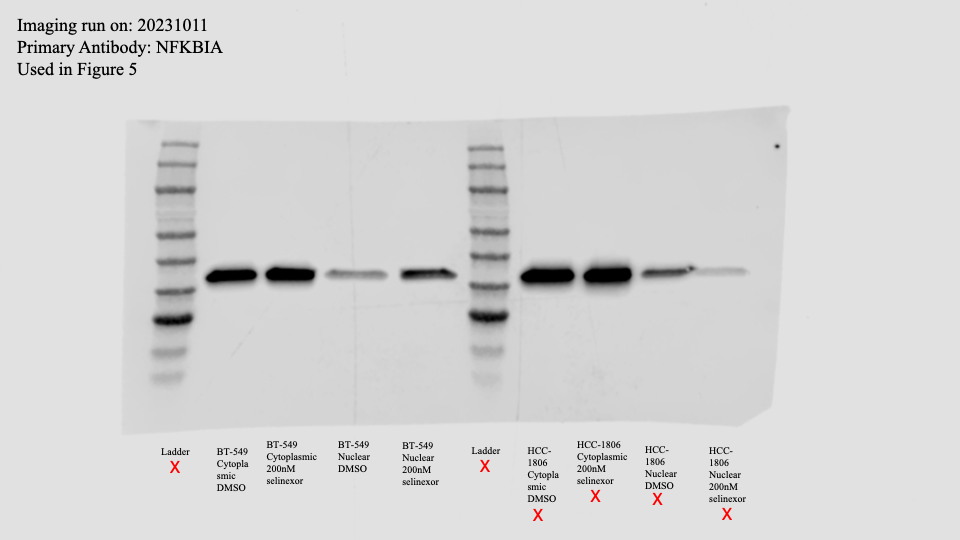

Supplement: Supplementary file 1 [file cancers-16-03980-s001.zip › westerns/DTNFKBIA-B.tiff]

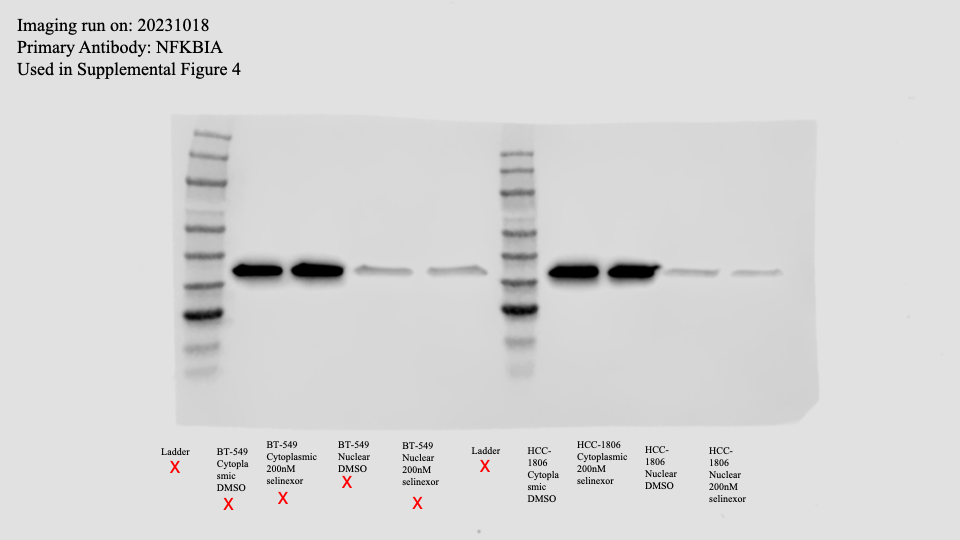

Supplement: Supplementary file 1 [file cancers-16-03980-s001.zip › westerns/DTNFKBIA-H.tiff]

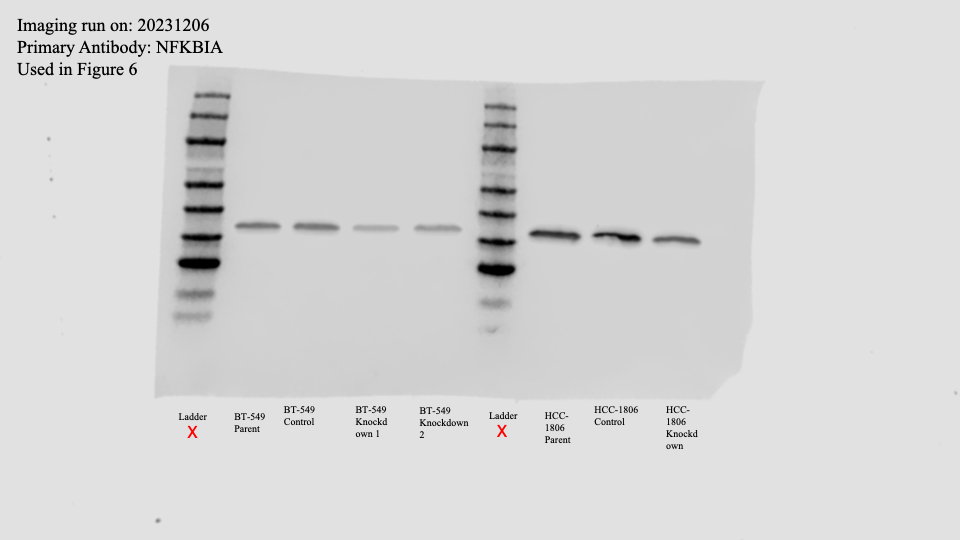

Supplement: Supplementary file 1 [file cancers-16-03980-s001.zip › westerns/NFKBIAKDB+H.tiff]

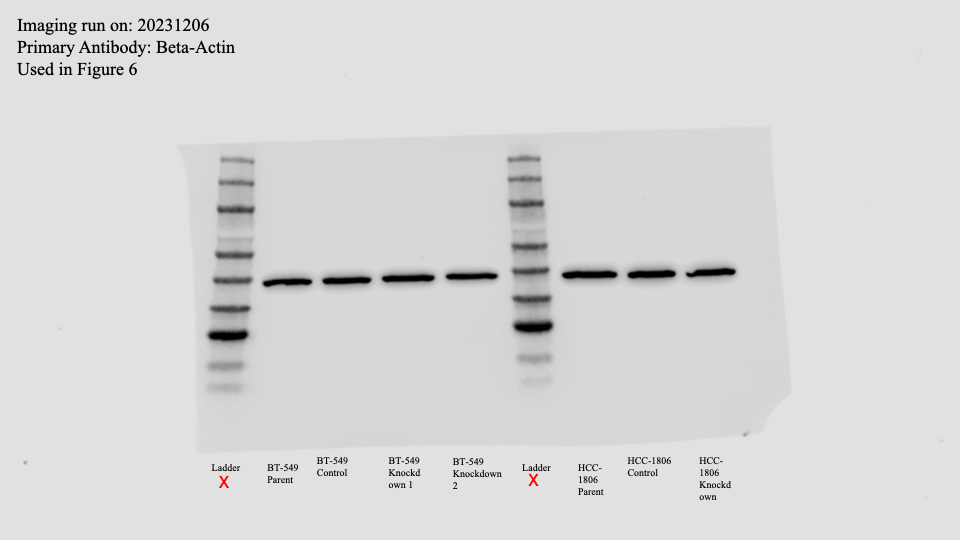

Supplement: Supplementary file 1 [file cancers-16-03980-s001.zip › westerns/NFKBIAKDBActinB+H.tiff]

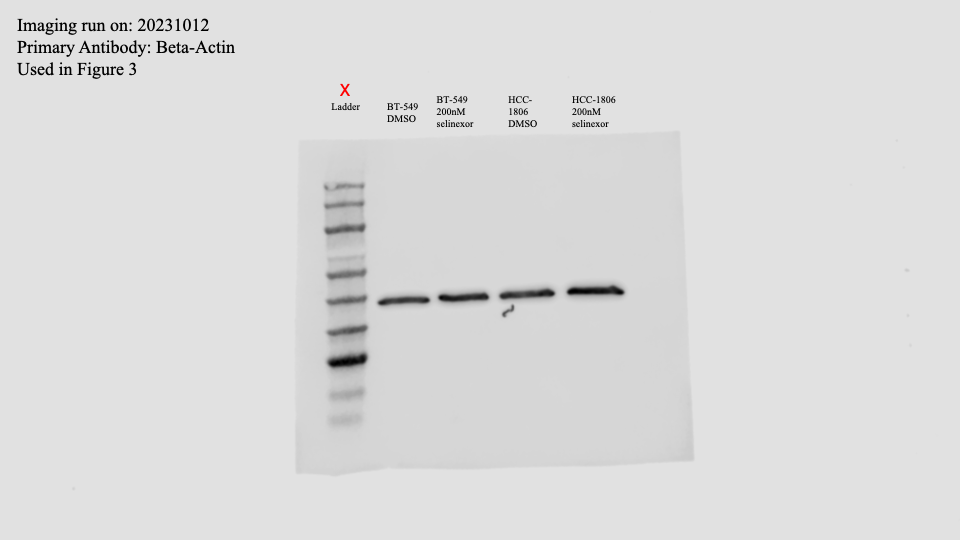

Supplement: Supplementary file 1 [file cancers-16-03980-s001.zip › westerns/WholeCellLysateBActinB+H.tiff]

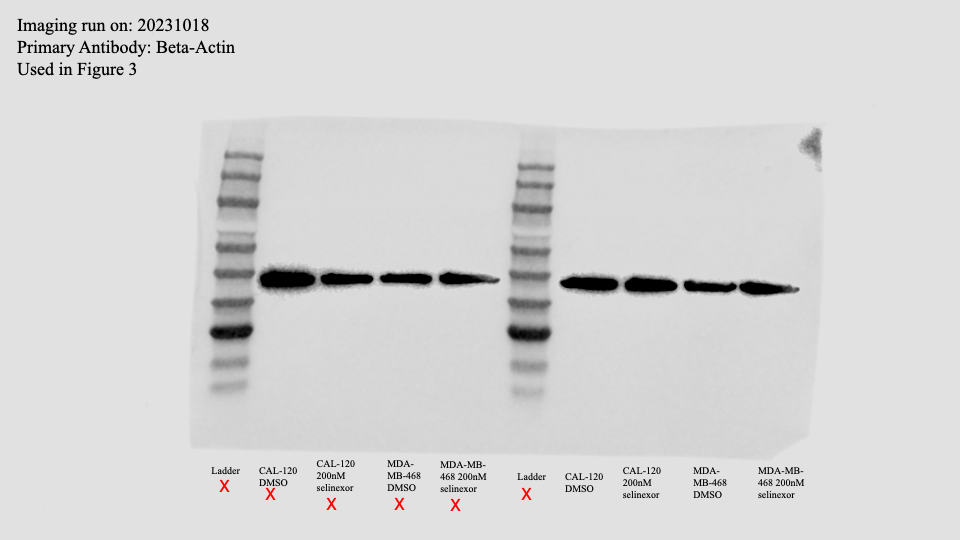

Supplement: Supplementary file 1 [file cancers-16-03980-s001.zip › westerns/WholeCellLysateBActinC+M.tiff]

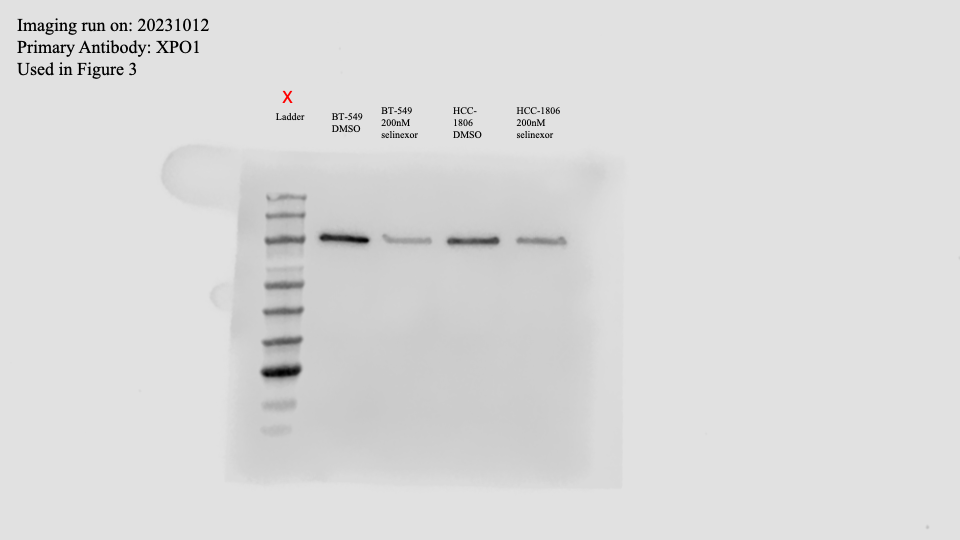

Supplement: Supplementary file 1 [file cancers-16-03980-s001.zip › westerns/WholeCellLysateXPO1B+H.tiff]

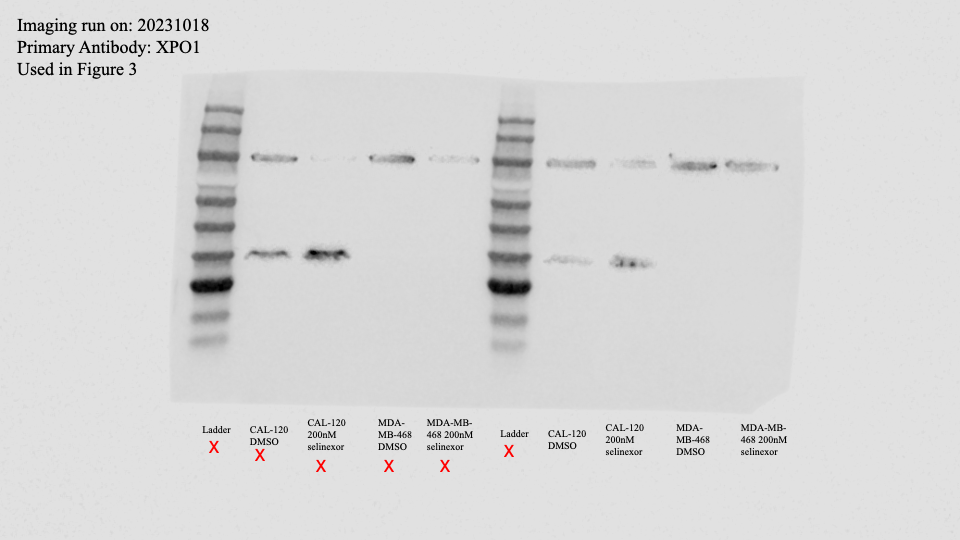

Supplement: Supplementary file 1 [file cancers-16-03980-s001.zip › westerns/WholeCellLysateXPO1C+M.tiff]
